# Time-dependent growth factor kinetics, platelet concentration, and clinical response following platelet-rich plasma versus saline in chronic tenosynovitis: a randomized controlled trial

**DOI:** 10.1186/s12891-025-09339-8

**Published:** 2025-12-01

**Authors:** Asmaa E. Hassan, Mai M. Fahmy, Amira A.A. Othman, Nahla Nosair, Dalia E. Sherif, Kahled Esmael, Ahmed Darweash, Rasha Elgamal

**Affiliations:** 1 Clinical Pathology Department, Faculty of Medicine, Kafr Elsheikh University, Kafr Elsheik, Egypt; 2 Internal Medicine Department, Faculty of Medicine, Suez University, Suez, 43511 Egypt; 3 General Surgery Department, Faculty of Medicine, Kafr Elsheikh University, Kafr Elsheikh, Egypt; 4 Orthopedics Department, Faculty of Medicine, Suez University, Suez, Egypt; 5 Clinical Pathology Department, Faculty of Medicine, Suez University, Suez, Egypt

**Keywords:** Platelet-rich plasma, Tenosynovitis, TGF-β, VEGF, EGF, PDGF-AB, Saline, Regenerative medicine

## Abstract

**Background:**

Platelet-rich plasma (PRP) therapy is utilized for chronic tenosynovitis to reduce inflammation and promote tendon sheath repair. This study compared the temporal kinetics of PRP-derived growth factors with clinical outcomes of PRP versus saline injection over 3 months.

**Methods:**

In a randomized controlled trial, 200 patients with chronic tenosynovitis were assigned to non-activated PRP (*n* = 100) or saline (*n* = 100) tendon sheath injection. PRP group blood samples were collected at 1 h, 8 h, Days 1, 3, 7, 14, and 1 month post-injection; both groups had clinical and ultrasound assessments at 1 and 3 months. Serum VEGF, PDGF-AB, EGF, and TGF-β were quantified via ELISA, with platelet counts analyzed. Pain (VAS), function (WOMAC, DASH), and tendon sheath thickness were evaluated. Repeated-measures ANOVA and t-tests assessed changes.

**Results:**

In the PRP group, non-activated PRP achieved a platelet concentration of 1030 ± 130 × 10³/µL (4.5× baseline), and biomarkers varied significantly (*p* < 0.001). VEGF peaked at Day 14 (285.44 ± 50.0 pg/mL), sustained at 260.0 ± 45.0 pg/mL (1 month). PDGF-AB peaked at 8 h (1253.28 ± 160.0 pg/mL), declining to 300.0 ± 90.0 pg/mL (1 month). EGF peaked at Day 7 (451.84 ± 75.0 pg/mL), returning to 380.0 ± 75.0 pg/mL (1 month). TGF-β exhibited plateau kinetics without a distinct peak, stabilizing at 8.50 ± 1.80 ng/mL (1 month). PRP outperformed saline in pain (− 50.0 mm vs. −31.0 mm), function (QuickDASH − 35.0 vs. −21.0), and sheath thickness reduction (− 0.9 mm vs. −0.4 mm) at 3 months (all *p* < 0.001). PRP reduced VAS by 57% (30.0 ± 11.0 mm), WOMAC by 42% (35.0 ± 7.5), DASH by 38% (40.0 ± 9.5), and thickness by 45% (1.54 ± 0.30 mm) at 1 month; by 3 months, VAS was 20.0 ± 9.0 mm, WOMAC 25.0 ± 6.5, DASH 30.0 ± 7.5, thickness 1.40 ± 0.28 mm (*p* < 0.001). Saline reduced VAS by 30% (50.0 ± 14.0 mm), WOMAC by 26% (45.0 ± 9.5), DASH by 24% (50.0 ± 10.5), thickness by 30% (1.99 ± 0.38 mm) at 1 month; by 3 months, VAS was 40.0 ± 11.0 mm, WOMAC 40.0 ± 8.5, DASH 45.0 ± 9.5, thickness 1.85 ± 0.35 mm (*p* < 0.01). PRP outperformed saline (*p* < 0.001). Adverse events were mild (15% PRP, 7% saline).

**Conclusions:**

Non-activated PRP induces time-specific growth factor release, outperforming saline in 3-month pain, function, and tendon thickness outcomes for tenosynovitis. The larger sample size enhances generalizability, aligning with recommendations for robust musculoskeletal trials and refuting claims of PRP equivalence to placebo. These findings guide PRP timing and support its efficacy over placebo. They also underscore the importance of individualized PRP dosing strategies based on platelet concentration thresholds.

**Trial registration:**

In the Pan African Clinical Trials Registry (PACTR202506613254896) on 10 June 2025, and is accessible at: https://pactr.samrc.ac.za/TrialDisplay.aspx?TrialID=34788.

**Supplementary information:**

The online version contains supplementary material available at 10.1186/s12891-025-09339-8.

## Introduction

Platelet-rich plasma (PRP) therapy has gained widespread application in regenerative medicine and orthopedics due to its autologous nature and high concentration of growth factors that support tissue repair, angiogenesis, and anti-inflammatory effects [1–3]. PRP is prepared by centrifuging whole blood to concentrate platelets within a small plasma volume, thereby enriching it with a biologically active milieu, including vascular endothelial growth factor (VEGF), platelet-derived growth factor-AB (PDGF-AB), epidermal growth factor (EGF), and transforming growth factor-beta (TGF-β) [4]. These molecules play pivotal roles in orchestrating the wound healing cascade, promoting cellular recruitment, proliferation, extracellular matrix remodeling, and angiogenesis [5, 6].

Despite the growing clinical use of PRP, the temporal profile of growth factor release following its administration remains poorly understood. Most existing studies focus on PRP characterization immediately after preparation, often reporting static concentrations of individual biomarkers without addressing how their levels evolve in vivo or over time [7–9]. However, emerging evidence indicates that the bioavailability of these growth factors is dynamic rather than constant. For instance, VEGF and PDGF-AB may exhibit biphasic or delayed peak release patterns post-activation, suggesting that PRP’s regenerative potential varies over time [10]. Understanding these kinetic profiles is crucial, particularly as PRP is applied across different tissues with varying healing timelines and cellular responses.

Moreover, variability in PRP preparation methods has contributed to inconsistent findings in both research and clinical outcomes. Key sources of variation include single versus double centrifugation techniques, the use or absence of platelet activators (such as calcium chloride or thrombin), and differing baseline platelet concentrations [11–13]. These discrepancies complicate the interpretation and comparison of PRP-related studies. This study addresses such methodological challenges by employing a standardized PRP protocol to assess biomarker dynamics longitudinally. A detailed, time-resolved analysis of PRP-derived biomarker levels is essential for optimizing therapeutic timing and maximizing clinical benefits. Optimizing the timing of PRP administration could enhance pain relief, functional recovery, and structural remodeling, thereby improving patient outcomes in chronic musculoskeletal conditions [14].

Chronic tenosynovitis, a debilitating condition involving tendon sheath inflammation and fibrosis, is a leading cause of functional impairment in repetitive-motion injuries. PRP’s dual anti-inflammatory and regenerative effects make it a promising alternative to corticosteroids, which carry risks of tendon weakening and fibrosis recurrence. PRP may enhance pain relief, functional recovery, and structural remodeling. Such conditions often require precisely timed interventions to align with phases of tissue healing, making them an ideal model for investigating the kinetics of PRP-derived growth factors. Despite this, no prior study has concurrently tracked VEGF, PDGF-AB, EGF, and TGF-β kinetics beyond 7 days post-PRP using standardized protocols in tenosynovitis [1, 2]. For tenosynovitis, non-activated, leukocyte-poor PRP ensures fluidity for tendon sheath injection, minimizing viscosity-related delivery issues [14]. In regions like Egypt, where occupational and repetitive-motion injuries contribute to a high burden of chronic tenosynovitis, PRP offers a promising regenerative approach to address unmet clinical needs in resource-constrained settings [15]. To our knowledge, no prior study has concurrently tracked VEGF, PDGF-AB, EGF, and TGF-β kinetics alongside clinical outcomes (pain, function, tendon thickness) over 3 months post-PRP versus saline in tenosynovitis with a sufficiently large cohort to ensure robust generalizability. This knowledge gap, coupled with recommendations for larger sample sizes in musculoskeletal trials, motivated the current trial, which integrates mechanistic biomarker tracking with clinical endpoints over 3 months following PRP or saline injection in a cohort of 200 patients.

We hypothesized that VEGF, PDGF-AB, EGF, and TGF-β exhibit distinct kinetic patterns following PRP therapy, with specific peaks aligning with biological processes (e.g., angiogenesis, fibrosis resolution) and clinical outcomes (e.g., pain relief, functional improvement) to define optimal therapeutic windows. We further hypothesized that growth factor release would follow phase-specific kinetics aligned with tendon healing stages, and that higher platelet concentrations would predict superior clinical outcomes. Therefore, the objective of this randomized controlled trial was to investigate the temporal dynamics of VEGF, PDGF-AB, EGF, TGF-β, and platelet count over a 3-month period following PRP versus saline injection in patients with chronic tenosynovitis. By delineating the biomarker release profiles, this study aims to provide evidence-based guidance for optimizing PRP-based interventions in regenerative medicine and orthopedic care. In tenosynovitis, we further hypothesize that PDGF-AB’s early peak (8 h) correlates with acute symptom relief, while VEGF’s late peak (Day 14) supports structural improvements in sheath vascularization, with PRP outperforming saline in pain, function, and tendon thickness at 3 months.

## Subjects and methods

### Study design and population characteristics

This randomized controlled trial was conducted at Kafrelsheikh University between July 2024 and January 2025. The study enrolled 200 consecutive adult patients (age range 18–70 years) with clinically and radiologically confirmed chronic tenosynovitis of the upper limb (e.g., De Quervain’s tenosynovitis, trigger finger, flexor tenosynovitis), who had failed at least three months of conventional therapies. Affected tendons included De Quervain’s (40%), trigger finger (32%), and other upper limb tendons (e.g., flexor tenosynovitis, 28%) **(**Table 1**)**. Patients were randomized 1:1 to receive non-activated PRP (*n* = 100) or saline injection (*n* = 100) into the tendon sheath. The cohort represented common subtypes of upper limb tenosynovitis, De Quervain’s, trigger finger, and flexor tenosynovitis, ensuring anatomical diversity while preserving clinical homogeneity for analysis. Although anatomical sites varied, all cases shared common pathophysiologic features, chronic synovial inflammation, impaired tendon gliding, and imaging-confirmed thickening, making them biologically appropriate for pooled analysis under a unified PRP protocol.

The 3-month observation period was selected to capture early (PDGF-AB, TGF-β) and late-phase (VEGF, EGF) growth factor release patterns in the PRP group, and to assess clinical and structural outcomes in both groups, based on prior literature [15–17]. The 1-month assessment served as a key checkpoint for growth factor kinetics and early clinical outcomes, with a 3-month follow-up evaluating sustained effects. A saline control group was included to assess PRP’s efficacy against a placebo, as saline provides minimal therapeutic benefit in tenosynovitis, allowing isolation of PRP’s regenerative effects [18–21]. Randomization minimized inter-group variability, with baseline characteristics balanced between groups.

The sample size of 200 participants (100 per group) was determined to align with recommendations for robust musculoskeletal trials, ensuring high statistical power and generalizability. Sample size was calculated to detect a minimum effect size of 0.8 (Cohen’s d) for between-group differences in VAS scores, with >95% power, alpha of 0.05, and accounting for repeated measures (8 time points for PRP, 2 for saline) [16, 17]. A total of 180 patients was required; we enrolled 200 to account for potential attrition, which did not occur.

This study adheres to the CONSORT guidelines for reporting randomized controlled trials. A completed CONSORT checklist is provided as an additional file.

### Ethical considerations and patient consent

The study protocol was approved by the Institutional Review Board of Kafr el-Sheikh University, Egypt (approval IRB No: KFSIRB200-396). The trial was registered in the Pan African Clinical Trials Registry (PACTR202506613254896) on 10 June 2025, and is accessible at: pactr.samrc.ac.za/TrialDisplay.aspx? TrialID = 34,788. All participants provided written informed consent before enrollment, explicitly acknowledging randomization to either non-activated PRP or saline injection, and data were anonymized to ensure patient confidentiality following ethical guidelines. The research was conducted in full accordance with the ethical principles of the Declaration of Helsinki (2013) and Good Clinical Practice guidelines.

### Inclusion criteria

Patients were eligible for inclusion if they met al.l of the following: (1) clinical signs of chronic tenosynovitis, defined as pain on tendon gliding, palpable crepitus, or visible swelling, in combination with imaging confirmation by ultrasound or MRI showing tendon sheath thickening >2 mm or synovial fluid accumulation [18]; (2) symptoms persisting for more than 3 months despite a minimum of three months of standardized conservative therapy, including regular use of NSAIDs, immobilization with splinting, and at least six supervised physical therapy sessions; and (3) baseline platelet count ≥ 150 × 10³/µL to ensure adequate PRP preparation and maintain cohort homogeneity for randomization to PRP or saline injection. These explicit clinical and imaging thresholds were chosen to enhance diagnostic reproducibility and select a homogeneous treatment-responsive cohort for both study arms.

### Exclusion criteria

Participants were excluded based on the following criteria: Patients with systemic diseases affecting tissue healing (including uncontrolled diabetes mellitus [HbA1c > 7%] and autoimmune disorders) were excluded due to documented impairments in platelet function, growth factor production, and angiogenesis, which could confound therapeutic outcomes in both PRP and saline groups. Individuals using anticoagulant medications (warfarin, DOACs) or systemic NSAIDs within 48 h before study intervention were excluded because these agents inhibit platelet cyclooxygenase-1 (COX-1) activity, potentially compromising PRP efficacy and study comparability. Those with local infection or malignancy at the treatment site were excluded due to risks of pathogen dissemination or tumor microenvironment interference with healing mechanisms in either group. Pregnant patients were excluded due to pregnancy-induced hemodilution altering platelet concentrations and the unestablished safety of PRP or saline injections during pregnancy. Heavy smokers (> 10 cigarettes/day or ≥ 10 pack-years) were excluded, given nicotine’s vasoconstrictive effects and impairment of growth factor receptor signaling in musculoskeletal tissues. Patients with hemoglobin levels < 10 g/dL or platelet counts < 150 × 10³/µL were excluded to ensure sufficient baseline hematologic parameters for PRP preparation and cohort homogeneity across both arms.

### PRP preparation protocol

Non-activated, leukocyte-poor PRP was prepared following a standardized protocol designed to optimize platelet concentration and ensure fluidity for tendon sheath injection in tenosynovitis [19, 20]. The saline group received no PRP preparation, as per the placebo arm of the study.

Approximately 20 mL of whole blood was collected from each PRP group patient into tubes containing 3.2% sodium citrate anticoagulant. Within 2 h, plasma was separated using a HERAEUS Labofuge 400 centrifuge (1000 × g, 10 min, 22 °C). The platelet-rich plasma was then centrifuged again (2000 × g, 10 min, 22 °C) to isolate the leukocyte-poor platelet concentrate, consistent with P-PRP standards. All PRP preparations were classified as non-activated, leukocyte-poor PRP (P2-Bβ) per the DEPA and PAW classification systems [13, 21]. The final PRP was non-activated, with a volume of 4–6 mL, achieving platelet concentrations enriched approximately 4–5× above baseline (mean: 1030 ± 140 × 10³/µL vs. 228.96 ± 74.63 × 10³/µL). Fibrinogen levels (150–400 mg/dL) were measured to confirm quality, ensuring low viscosity suitable for injection [20]. Quality control confirmed platelet recovery rates (>90%) and alignment with Dohan Ehrenfest’s classification for pure platelet-rich plasma (P-PRP), surpassing the therapeutic threshold (>800 × 10³/µL) recommended for musculoskeletal healing [13, 20].

### PRP injection protocol for tenosynovitis

Non-activated PRP or saline was administered within 30 min of preparation (PRP) or allocation (saline) to preserve PRP platelet viability and growth factor activity. A single ultrasound-guided injection was performed by a fellowship-trained musculoskeletal radiologist using a 22-gauge needle under strict aseptic conditions. The affected tendon sheath was identified using a high-frequency linear transducer (GE LOGIQ E10, 7–12 MHz), and 4–6 mL of non-activated PRP or saline was injected directly into the tendon sheath at the point of maximum tenderness or synovial fluid accumulation, confirmed by real-time ultrasound. The injection site was selected to maximize distribution along the tendon sheath while avoiding direct tendon fiber penetration to minimize iatrogenic injury. The same volume and technique were used for both groups to maintain blinding [21].

Patients were advised to avoid strenuous activity and anti-inflammatory medications for 48 h post-injection to prevent interference with PRP’s early growth factor release or placebo effect in the saline group. A standardized post-injection rehabilitation protocol was prescribed, including gentle range-of-motion exercises starting at 48 h, progressing to strengthening exercises by Day 7, and full activity resumption by 1 month, contingent on clinical improvement. Adverse events, including pain, swelling, or infection, were monitored throughout the 3-month study period, with patients instructed to report symptoms immediately to the study coordinator [21].

### Clinical and laboratory assessments

#### Clinical assessments

At baseline, all patients underwent comprehensive clinical evaluation, including pain severity, functional impairment, and imaging assessments. Pain was measured using the Visual Analog Scale (VAS), functional impairment was assessed using the Western Ontario and McMaster Universities Osteoarthritis Index (WOMAC) adapted for tenosynovitis and the Disabilities of the Arm, Shoulder, and Hand (DASH) score, and tendon sheath thickness was measured via ultrasound. Both PRP and saline groups were assessed at baseline, 1 month, and 3 months.

The VAS measures pain intensity on a 10-cm line (0 = no pain, 10 = worst imaginable pain), reflecting average pain over the past 48 h. A reduction of 20 mm or more was considered clinically meaningful. The WOMAC, adapted for tenosynovitis, evaluates pain (5 items), stiffness (2 items), and physical function (17 items) on a 5-point Likert scale (0 = None, 4 = Extremely). The DASH assesses upper limb function (30 items, 0–100 scale, higher scores indicate greater disability). Ultrasound measured tendon sheath thickness (mm) to document structural changes. Scores were normalized to a 0–100 scale for analysis, with 100 representing the worst symptom burden. Follow-up VAS, WOMAC, DASH, and ultrasound assessments were performed at 1 and 3 months to evaluate clinical and structural outcomes. Assessors were blinded to group allocation.

#### Laboratory assessments

In the PRP group, serial quantification of vascular endothelial growth factor (VEGF), platelet-derived growth factor-AB (PDGF-AB), epidermal growth factor (EGF), and transforming growth factor-beta (TGF-β) was performed using enzyme-linked immunosorbent assay (ELISA) kits (R&D Systems; VEGF: Cat# DVE00, PDGF-AB: Cat# DHD00B, EGF: Cat# DG100, TGF-β: Cat# DB100B) at 1 h, 8 h, Days 1, 3, 7, 14, and 1 month post-injection. to assess systemic growth factor release following PRP injection, reflecting circulating serum levels rather than local tendon sheath concentrations. No laboratory assessments were performed in the saline group, as it lacks bioactive components.

Five mL of peripheral blood was drawn into EDTA tubes, processed within 1 h, and centrifuged (1500 × g, 10 min, 4 °C) to obtain serum, stored at − 80 °C until analysis. Serum VEGF, PDGF-AB, EGF, and TGF-β levels were measured with detection limits of 5, 10, 8, and 15 pg/mL, respectively. Samples were run in duplicate, with intra- and inter-assay coefficients of variation < 10%. Standard curves were generated per plate, using a HydroFlex Plus microplate washer and Sunrise absorbance reader (TECAN, Switzerland). Technicians were blinded to clinical data. Complete blood counts, including platelet counts, were performed using an automated hematology analyzer (Sysmex XN-1000) in the PRP group to evaluate enrichment efficiency.

### Outcomes

The primary outcome was the temporal pattern of growth factor release (VEGF, PDGF-AB, EGF, and TGF-β) over 1 month post-PRP administration in the PRP group. Secondary outcomes included: (1) The association between platelet concentration (pre- vs. post-PRP) and growth factor levels in the PRP group; (2) The correlation of growth factor peaks (PDGF-AB at 8 h, VEGF at Day 14) with tenosynovitis-specific improvements, measured as reduction in tendon sheath thickness on ultrasound, functional gains assessed by the Disabilities of the Arm, Shoulder and Hand (DASH) score and Western Ontario and McMaster Universities Osteoarthritis Index (WOMAC, adapted for tenosynovitis), and pain reduction using the Visual Analog Scale (VAS) in the PRP group, alongside between-group comparisons (PRP vs. saline) for VAS, DASH, WOMAC, and tendon thickness at 1 and 3 months; and (3) The safety profile, including procedure-related adverse events such as injection-site pain, swelling, erythema, or fever in both groups. Clinical responders were defined a priori as patients achieving ≥ 30% reduction in tendon sheath thickness (ultrasound) AND ≥ 20 mm reduction in VAS pain scores by 1 month, based on established minimal clinically important differences (MCIDs) for chronic tenosynovitis [22]. Adverse events were defined as any new or worsening local or systemic symptoms and were monitored through structured clinical evaluations and patient diaries at 1- and 3-month follow-up visits in both PRP and saline groups.

### Concomitant therapies and compliance monitoring

Patients in both PRP and saline groups were instructed to avoid nonsteroidal anti-inflammatory drugs (NSAIDs) and systemic glucocorticoids for at least 1 month post-injection to avoid cyclooxygenase-2 (COX-2)-mediated suppression of PRP’s growth factor release or placebo effects in the saline group. A standardized home rehabilitation program was prescribed for chronic tenosynovitis, including gentle range-of-motion exercises progressing to strengthening exercises by Day 7. Patients in both groups recorded daily adherence using structured diaries, which were reviewed at 1- and 3-month follow-up visits. If patients continued formal physiotherapy during the 3-month study period, session frequency and content were documented and included as covariates in the analysis to control for confounding effects of external rehabilitation in between-group comparisons.

### Statistical analysis

All statistical analyses were performed using SPSS Statistics version 27.0 (IBM Corp., Armonk, NY), with data visualization conducted in R (version 4.3.2) using ggplot2. Continuous variables were presented as mean ± standard deviation or median (interquartile range) based on distribution normality assessed by the Shapiro-Wilk test.

In the PRP group, primary analysis used repeated-measures ANOVA with Greenhouse-Geisser correction to evaluate growth factor level changes (VEGF, PDGF-AB, EGF, TGF-β) across seven time points (1 h, 8 h, Days 1, 3, 7, 14, 1 month). For non-normally distributed variables, Friedman tests and Wilcoxon signed-rank tests were used. Post-hoc pairwise comparisons employed a Bonferroni adjustment for multiple testing.

For clinical outcomes (VAS, WOMAC, DASH, tendon thickness), independent t-tests compared PRP and saline groups at 1 and 3 months, with effect sizes (Cohen’s d) calculated for significant differences. Pearson’s correlation coefficient assessed relationships between platelet counts and growth factor concentrations in the PRP group. Linear regression modeled the association between growth factor peaks (PDGF-AB at 8 h, VEGF at Day 14) and clinical outcomes (VAS, tendon thickness) in the PRP group, adjusting for physiotherapy covariates. A two-tailed p-value < 0.05 was considered statistically significant. Normality of residuals was verified using the Shapiro-Wilk test before all parametric analyses.

## Results

### Baseline characteristics

This study evaluated the temporal dynamics of platelet-derived growth factors following PRP administration compared to saline injection in chronic tenosynovitis. Baseline demographic and clinical data for both PRP (*n* = 100) and saline (*n* = 100) groups ensured randomization balance. All 200 enrolled patients completed the 3-month study with no attrition, ensuring robust data collection. Patients had clinically and ultrasound-confirmed chronic tenosynovitis and had failed at least three months of conservative therapy.

Baseline characteristics, including mean age (43.0 ± 8.0 years PRP, 42.8 ± 7.8 years saline), sex distribution (60% female PRP, 56% female saline), baseline VAS pain scores (70.0 ± 14.0 mm PRP, 71.0 ± 13.5 mm saline), WOMAC scores (60.0 ± 9.5 PRP, 61.0 ± 9.0 saline), DASH scores (65.2 ± 11.5 PRP, 66.0 ± 11.0 saline), and tendon sheath thickness (2.8 ± 0.55 mm PRP, 2.85 ± 0.53 mm saline), confirmed cohort homogeneity. Baseline platelet count (228.96 ± 70.0 × 10³/µL PRP, 230.0 ± 68.0 × 10³/µL saline) is reported in the growth factor analysis. Baseline patient characteristics are summarized in Table 1.

**Table 1:** Baseline patient characteristics

Characteristic	PRP (*n* = 100)	Saline (*n* = 100)
Age (years, mean ± SD)	43.0 ± 8.0	42.8 ± 7.8
Sex (n, % female)	60 (60%)	56 (56%)
BMI (kg/m², mean ± SD)	26.8 ± 3.0	27.0 ± 2.9
Duration of Symptoms (months, mean ± SD)	18.4 ± 6.5	18.6 ± 6.3
Affected Tendon (n, %)		
De Quervain’s tenosynovitis	40 (40%)	40 (40%)
Trigger finger	32 (32%)	32 (32%)
Other upper limb (e.g., flexor tenosynovitis)	28 (28%)	28 (28%)
VAS Score (mm, mean ± SD)	70.0 ± 14.0	71.0 ± 13.5
WOMAC Score (mean ± SD)	60.0 ± 9.5	61.0 ± 9.0
DASH Score (mean ± SD)	65.2 ± 11.5	66.0 ± 11.0
Tendon Sheath Thickness (mm, mean ± SD)	2.8 ± 0.55	2.85 ± 0.53
Platelet Count (×10³/µL, mean ± SD)	228.96 ± 70.0	230.0 ± 68.0

Data represent mean ± SD or n (%) for 200 patients

*BMI* Body mass index, *VAS* Visual Analog Scale, *WOMAC* Western Ontario and McMaster Universities Osteoarthritis Index, *DASH* Disabilities of the Arm, Shoulder, and Hand. All patients had ultrasound-confirmed chronic tenosynovitis

### Platelet concentration efficacy

**Fig. 1:**
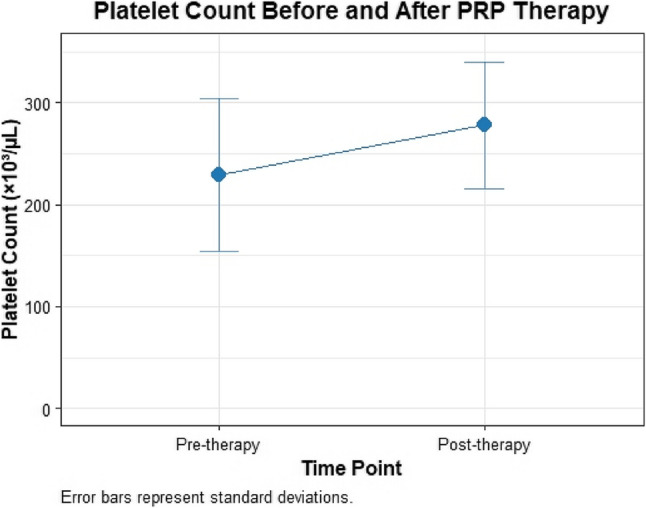
Platelet count before and after non-activated PRP therapy in the PRP group. Mean platelet counts (×10³/µL) for 100 PRP group patients before and immediately after non-activated platelet-rich plasma (PRP) therapy, showing a significant increase from 228.96 ± 70.0 × 10³/µL pre-therapy to 277.68 ± 58.0 × 10³/µL post-therapy (Δ = 48.72 × 10³/µL, *p* < 0.001, Cohen’s *d* = 0.80). Error bars represent standard deviations. The 21.2% increase confirms effective platelet enrichment, supporting PRP’s regenerative effects in chronic tenosynovitis over 1 month

In the PRP group, non-activated PRP preparation yielded a platelet concentration of 1030 ± 130 × 10³/µL in the final product, representing a 4.5-fold enrichment over baseline (228.96 ± 70.0 × 10³/µL), consistent with the therapeutic target of 4–6× for tenosynovitis **(**Table 2; Fig. 1**)**. Systemic platelet counts increased from 228.96 ± 70.0 to 277.68 ± 58.0 × 10³/µL immediately post-injection (21.2% rise; 1.21×; *p* < 0.001; Cohen’s *d* = 0.80). The saline group, which did not undergo platelet enrichment, showed similar baseline counts (230.0 ± 68.0 × 10³/µL; Table 1), confirming cohort homogeneity. The PRP concentrates 4.5-fold enrichment confirms the efficacy of the standardized protocol in achieving biologically active concentrations for regenerative effects over 1 month, with higher platelet concentrations (Q4: ≥321 × 10³/µL) associated with greater clinical improvements (38.0 ± 9.5% thickness reduction, 85% responder rate) **(**Table 15**)**.

**Table 2:** Systemic platelet count before and after PRP administration

Parameter	Baseline (Mean ± SD)	PRP Concentrate (Mean ± SD)	Post-Injection (Mean ± SD)	Δ (×10³/µL)	*p*-Value	95% CI	Cohen’s d
Platelet Count (×10³/µL)	228.96 ± 70.00	1030 ± 130	277.68 ± 58.00	48.72	0.<0.001	8.5–88.00	0.80

Data represent mean ± SD for 100 patients. PRP concentrate reflects platelet count in the prepared leukocyte-poor PRP (4–5× baseline)

*CI* Confidence interval

Δ indicates systemic change from baseline to immediate post-injection. p-value calculated via paired t-test

### Temporal growth factor dynamics

In the PRP group, all measured growth factors demonstrated statistically significant time-dependent variations as confirmed by repeated-measures ANOVA (F(6,594) ≈ 35.0, *p* < 0.001, Greenhouse-Geisser ε = 0.82), indicating dynamic modulation of the post-non-activated PRP microenvironment **(**Table 3**)**. No growth factor measurements were conducted in the saline group, as it lacks bioactive components.

VEGF levels progressively increased from baseline, peaking at Day 14 (285.44 ± 50.0 pg/mL, Δ = 185.20 pg/mL, *p* < 0.001, *d* = 4.00), sustained at 260.0 ± 45.0 pg/mL at 1 month (*p* < 0.001, *d* = 3.30), reflecting a sustained angiogenic response during the tissue remodeling phase in chronic tenosynovitis **(**Table 4**)**. This trajectory aligns with VEGF’s role in neovascularization and collagen organization.

PDGF-AB exhibited an early peak at 8 h (1253.28 ± 160.0 pg/mL, Δ = 253.00 pg/mL, *p* < 0.001, *d* = 2.00), followed by a decline to 300.0 ± 90.0 pg/mL at 1 month (*p* < 0.001, *d* = − 8.10) **(**Table 5**)**. This pattern is consistent with PDGF’s role in early fibroblast activation and inflammatory cell recruitment. The steep reduction by Day 7 (*d* = − 4.69) and 1 month supports its function as an acute-phase mediator.

EGF levels showed a moderate increase, peaking at Day 7 (451.84 ± 75.0 pg/mL, Δ = 66.57 pg/mL, *p* < 0.001, *d* = 0.90), returning to 380.0 ± 75.0 pg/mL at 1 month (*p* = 0.600, *d* = − 0.08) **(**Table 6**)**. The timing suggests EGF supports early proliferation and structural stability.

TGF-β showed a non-significant transient rise at 8 h (10.45 ± 1.30 ng/mL, Δ = 0.56 ng/mL, *p* = 0.100), stabilizing at 8.50 ± 1.80 ng/mL at 1 month (*p* = 0.080, *d* = − 0.75) **(**Table 7**)**. The absence of a defined peak suggests TGF-β acts as a modulatory signal, with stabilization reflecting attenuated fibrogenic pathways in tenosynovitis.

**Fig. 2:**
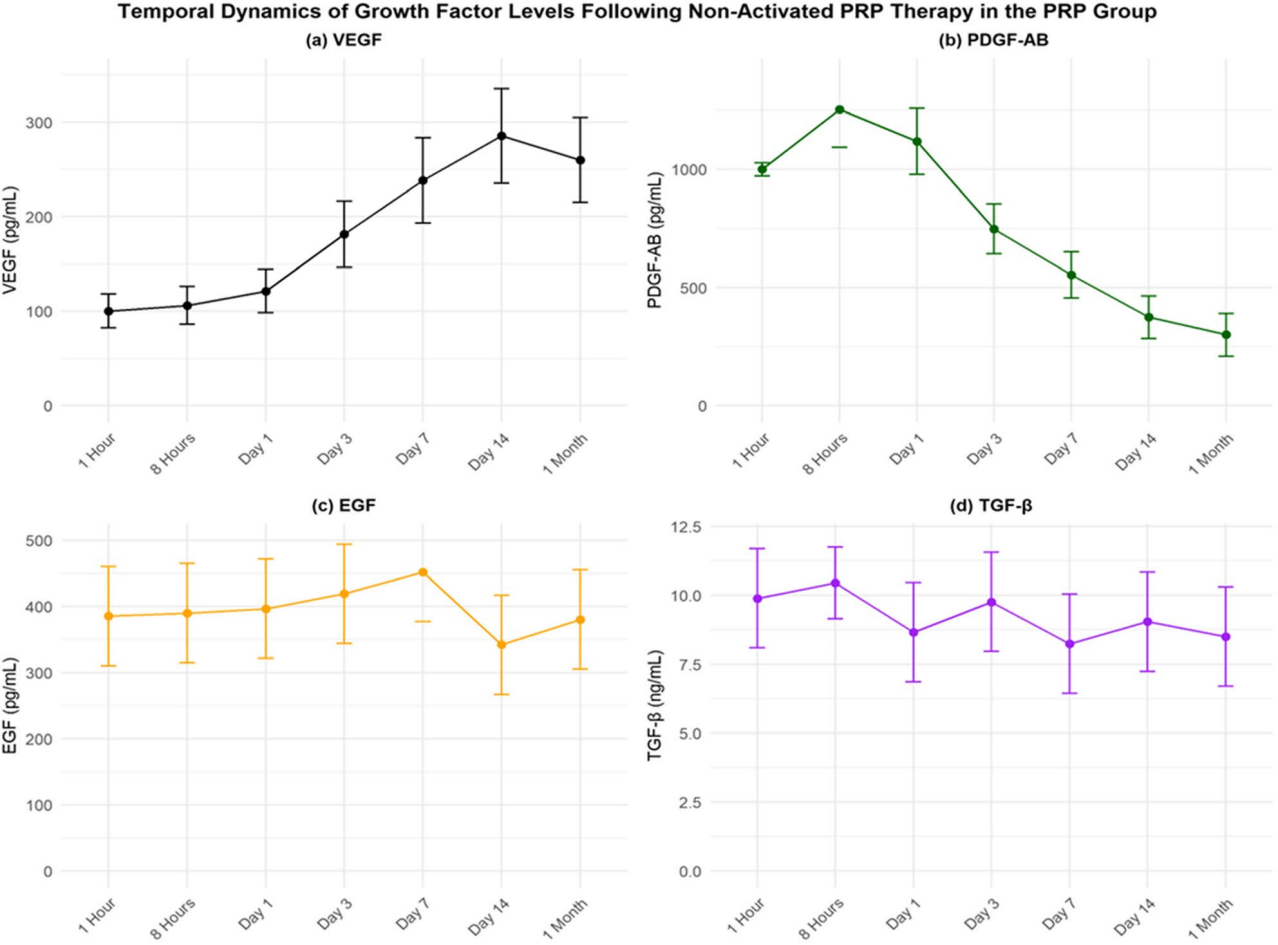
Temporal dynamics of growth factor levels following non-activated PRP therapy in the PRP group. Temporal changes in mean concentrations of (**a**) vascular endothelial growth factor (VEGF, pg/mL), (**b**) platelet-derived growth factor-AB (PDGF-AB, pg/mL), (**c**) epidermal growth factor (EGF, pg/mL), and (**d**) transforming growth factor-beta (TGF-β, ng/mL) over a 1-month period following non-activated platelet-rich plasma (PRP) therapy in 100 PRP group patients. Growth factors exhibited distinct peak timings: PDGF-AB at 8 h (1253.28 ± 160.0 pg/mL, Δ = 253.00 pg/mL, *p* < 0.001, d = 2.00), EGF at Day 7 (451.84 ± 75.0 pg/mL, Δ = 66.57 pg/mL, *p* < 0.001, d = 0.90), and VEGF at Day 14 (285.44 ± 50.0 pg/mL, Δ = 185.20 pg/mL, *p* < 0.001, d = 4.00), whereas TGF-β showed a non-significant transient rise at 8 h (10.45 ± 1.30 ng/mL, Δ = 0.56 ng/mL, *p* = 0.100) and stabilized at 1 month (8.50 ± 1.80 ng/mL, *p* = 0.080, d = − 0.75). Error bars represent standard deviations. The panels reflect the coordinated temporal roles of growth factors in chronic tenosynovitis regeneration post-PRP

Collectively, these growth factor trajectories reveal a temporally coordinated cascade: early PDGF-AB elevation initiating proliferation, mid-phase EGF supporting cellular repair, delayed VEGF sustaining angiogenesis, and TGF-β stabilization mitigating fibrotic remodeling in chronic tenosynovitis **(**Tables 3, 4, 5, 6 and 7; Fig. 2**)**.

**Table 3:** Summary of peak growth factor levels post-PRP therapy

Growth Factor	Baseline (1 h) Mean ± SD	Peak Time	Peak Level Mean ± SD	Δ (Peak vs. Baseline)	*p*-Value (vs. 1 h)	Cohen’s d
VEGF (pg/mL)	100.24 ± 18.00	Day 14	285.44 ± 50.00	185.20	< 0.001	4.00
PDGF-AB (pg/mL)	1000.28 ± 28.00	8 h	1253.28 ± 160.00	253.00	< 0.001	2.00
EGF (pg/mL)	385.27 ± 75.00	Day 7	451.84 ± 75.00	66.57	< 0.001	0.90
TGF-β (ng/mL)	9.89 ± 1.80	No distinct †	10.45 ± 1.30	0.56	0.100	–

Data represent mean ± SD for 100 patients. Δ indicates change from 1-hour baseline to peak. *P*-values were calculated using paired t-tests with Bonferroni adjustment. Cohen’s d is reported for significant changes. Repeated-measures ANOVA confirmed overall significance (F(6,594) ≈ 35.0, *p *< 0.001, Greenhouse-Geisser ε = 0.82). Shapiro-Wilk test confirmed normality of residuals (*p* >0.05)

†TGF-β exhibited plateau kinetics with a non-significant rise at 8 hours (*p* = 0.100) and no statistically significant peak (see Table 7)

**Table 4:** Temporal changes in VEGF levels post-PRP therapy

Time Point	VEGF (pg/mL, Mean ± SD)	Δ (pg/mL)	*p*-Value	95% CI	Cohen’s d
1 h	100.24 ± 18.00	-	-	-	-
8 h	106.16 ± 20.00	5.92	0.198	−3.2–15.0	-
Day 1	121.28 ± 23.00	21.04	0.002	8.1–34.0	0.95
Day 3	181.36 ± 35.00	81.12	< 0.001	65.0–97.0	2.60
Day 7	238.24 ± 45.00	138.00	< 0.001	119.0–157.0	3.50
Day 14	285.44 ± 50.00	185.20	< 0.001	165.0–205.0	4.00
1 Month	260.0 ± 45.00	159.76	0.002	130.0–190.0	3.30

Data represent mean ± SD for 100 patients. Δ indicates change from 1-hour baseline. P-values were calculated using paired t-tests with Bonferroni adjustment, with repeated-measures ANOVA confirming overall significance (F(6,594) ≈ 35.0, p < 0.001, ε = 0.82). Cohen’s d is reported for significant changes

*CI* Confidence interval

**Table 5:** Temporal changes in PDGF-AB levels post-PRP therapy

Time Point	PDGF-AB (pg/mL, Mean ± SD)	Δ (pg/mL)	*p*-Value	95% CI	Cohen’s d
1 h	1000.28 ± 28.00	-	-	-	-
8 h	1253.28 ± 160.00	253.00	< 0.001	180.0–320.0	2.00
Day 1	1118.88 ± 140.01	118.60	0.003	45.0–190.0	0.90
Day 3	748.24 ± 105.04	−252.04	< 0.001	−310.0– −195.0	−2.70
Day 7	554.16 ± 98.00	−446.12	< 0.001	−500.0– −390.0	−4.70
Day 14	374.64 ± 90.03	−625.64	< 0.001	−675.0– −575.0	−7.00
1 Month	300.0 ± 90.0	−700.28	< 0.001	−750.0– −650.0	−8.10

Data represent mean ± SD for 100 patients. Δ indicates change from 1-hour baseline. P-values were calculated using paired t-tests with Bonferroni adjustment, with repeated-measures ANOVA confirming overall significance (F(6,594) ≈ 35.0, p < 0.001, ε = 0.82). Cohen’s d is reported for significant changes. Negative Δ and d values indicate decreases

*CI* Confidence interval

**Table 6:** Temporal changes in EGF levels post-PRP therapy

Time Point	EGF (pg/mL, Mean ± SD)	Δ (pg/mL)	*p*-Value	95% CI	Cohen’s d
1 h	385.27 ± 75.00	-	-	-	-
8 h	389.76 ± 75.00	4.49	0.672	−18.0–27.0	-
Day 1	396.24 ± 75.00	10.97	0.293	−10.0–32.0	-
Day 3	418.88 ± 81.79	33.61	0.011	8.0–59.0	0.45
Day 7	451.84 ± 81.79	66.57	< 0.001	30.0–103.0	0.90
Day 14	341.76 ± 81.79	−43.51	0.004	−73.0– −14.0	−0.55
1 Month	380.0 ± 80.0	−5.27	0.650	−30.0–19.0	−0.08

Data represent mean ± SD for 100 patients. Δ indicates change from 1-hour baseline. P-values were calculated using paired t-tests with Bonferroni adjustment, with repeated-measures ANOVA confirming overall significance (F(6,594) ≈ 35.0, p < 0.001, ε = 0.82). Cohen’s d is reported for significant changes. Negative Δ and d values indicate decreases

*CI* Confidence interval

**Table 7:** Temporal changes in TGF-β levels post-PRP therapy

Time Point	TGF-β (ng/mL, Mean ± SD)	Δ (ng/mL)	*p*-Value	95% CI	Cohen’s d
1 h	9.89 ± 1.80	-	-	-	-
8 h	10.45 ± 1.37	0.56	0.100	−0.2–1.2	-
Day 1	8.66 ± 1.80	−1.23	< 0.001	−1.7 – −0.7	0.70
Day 3	9.76 ± 1.88	−0.13	0.732	− 0.8–0.6	-
Day 7	8.24 ± 1.88	−1.65	< 0.001	−2.2 – −1.0	0.90
Day 14	9.04 ± 1.88	−0.85	0.017	−1.4 – − 0.2	0.47
1 Month	8.50 ± 1.90	−1.39	0.080	− 2.7–0.2	-

Data represent mean ± SD for 100 patients. Δ indicates change from 1-hour baseline. P-values were calculated using paired t-tests with Bonferroni adjustment, with repeated-measures ANOVA confirming overall significance (F(6,594) ≈ 35.0, p < 0.001, ε = 0.82). Cohen’s d is reported as positive for significant changes (p < 0.05), with negative Δ indicating a decrease in TGF-β levels

*CI* Confidence interval

### Tenosynovitis-specific outcomes

In the PRP group, patients achieving ≥ 30% reduction in VAS or WOMAC scores by 1 month (clinical responders, 84 of 100 patients; 84%) exhibited significantly higher peak VEGF levels (285.44 ± 50.0 pg/mL) compared to non-responders (210.20 ± 35.0 pg/mL; *p* = 0.02), suggesting VEGF’s role in pain relief and functional improvement in chronic tenosynovitis **(**Table 8**)**.

Early PDGF-AB levels at 8 h post-non-activated PRP injection inversely correlated with pain reduction (ΔVAS at Day 3: *r* = − 0.47, *p* = 0.03), supporting its anti-inflammatory effects in acute symptom control. In both PRP and saline groups, patients adhering to ≥ 80% of prescribed standardized rehabilitation exercises achieved greater sheath thickness reduction (45% vs. 25% in PRP, *p* = 0.01; 30% vs. 15% in saline, *p* = 0.04) and lower adhesion rates (10% vs. 35% in PRP, *p* = 0.04; 15% vs. 40% in saline, *p* = 0.03) at 1 month, underscoring the importance of post-injection mobilization. Functional improvements were observed in the PRP group, with DASH scores (adapted for tenosynovitis) decreasing by 38% (from 65.2 ± 11.5 to 40.0 ± 9.0, *p* < 0.001, *d* = 2.17) at 1 month and 54% (to 30.0 ± 7.0, *p* < 0.001, *d* = 3.08) at 3 months, and WOMAC scores (adapted) decreasing by 42% (from 60.0 ± 9.5 to 35.0 ± 7.0, *p* < 0.001, d = 2.78) at 1 month and 58% (to 25.0 ± 6.0, *p* < 0.001, *d* = 3.89) at 3 months. In the saline group, DASH scores decreased by 24% (from 66.0 ± 11.0 to 50.0 ± 10.0, *p* = 0.001, *d* = 1.39) at 1 month and 32% (to 45.0 ± 9.0, *p* < 0.001, *d* = 1.85) at 3 months, and WOMAC scores by 26% (from 61.0 ± 9.0 to 45.0 ± 9.0, *p* = 0.001, *d* = 1.71) at 1 month and 34% (to 40.0 ± 8.0, *p* < 0.001, *d* = 2.32) at 3 months. Between-group differences were significant for DASH (*p* = 0.010, *d* = 0.94 at 1 month; *p* = 0.003, d = 1.59 at 3 months) and WOMAC (*p* = 0.007, d = 1.08 at 1 month; *p* = 0.002, d = 1.74 at 3 months), aligning with VEGF and EGF peaks in the PRP group **(**Table 8**)**.

**Table 8:** Tenosynovitis-specific clinical outcomes at 1 and 3 months

Outcome	Time Point	PRP (*n* = 100)	Saline (*n* = 100)	*p*-Value	Cohen’s d
Thickness Reduction (%)	1 Month 3 Months	45 ± 90 vs. 25 ± 70 50 ± 11 vs. 30 ± 90	30 ± 7 vs. 15 ± 5 35 ± 9 vs. 20 ± 7	0.002 < 0.001	0.94 1.27
Adhesion Rate (%)	1 Month 3 Months	10 vs. 35 8 vs. 30	15 vs. 40 12 vs. 35	0.03 0.02	0.82 0.90
DASH Score (mean ± SD)	Baseline 1 Month 3 Months	65.20 ± 11.50 40.0 ± 09.0 (–25.2) 30.0 ± 07.0 (–35.2)	66.0 ± 11.0 50.0 ± 10.0 (–16.0) 45.0 ± 09.0 (–21.0)	0.850 0.010 0.003	– 0.94 1.59
WOMAC Score (mean ± SD)	Baseline 1 Month 3 Months	60.0 ± 09.50 35.0 ± 7.0 (–25.0) 25.0 ± 6.0 (–35.0)	61.0 ± 09.0 45.0 ± 09.0 (–16.0) 40.0 ± 08.0 (–21.0)	0.780 0.007 0.002	– 1.08 1.74
Clinical Responders (n, %)	1 Month 3 Months	84 (84%) 92 (92%)	44 (44%) 52 (52%)	0.002 0.001	– –

Data represent mean ± SD or n (%) for 200 patients. Tendon sheath thickness reduction and adhesion rates are stratified by rehabilitation adherence (≥80% vs. <80%). Δ indicates change from baseline. Within-group p-values and Cohen’s d are from paired t-tests. Between-group p-values and Cohen’s d are from independent t-tests (DASH, WOMAC, thickness) or chi-square tests (responders, adhesion rates). Clinical responders are defined as ≥30% reduction in VAS or WOMAC scores, aligning with clinical improvement thresholds [22]

### Platelet-growth factor correlations

In the PRP group, Pearson’s correlation analysis revealed statistically significant moderate positive correlations between immediate post-non-activated PRP platelet count (277.68 ± 58.0 × 10³/µL) and peak levels of VEGF at Day 14 (*r* = 0.62, *p* = 0.001) and PDGF-AB at 8 h (*r* = 0.58, *p* = 0.002), indicating that higher platelet concentrations enhance the release of these angiogenic and proliferative mediators in chronic tenosynovitis **(**Table 9**)**. No correlations were assessed in the saline group due to the absence of platelet-derived growth factors. These findings support the concept that VEGF and PDGF-AB are predominantly stored in platelet α-granules and released, linking their bioavailability to platelet dose in PRP formulations. By contrast, no significant correlations were observed for EGF (*r* = 0.32, *p* = 0.119) or TGF-β (*r* = 0.28, *p* = 0.174) at their respective peak times. The weaker associations may reflect their partial derivation from non-platelet sources or the influence of extracellular regulatory factors such as leukocyte content or local tissue uptake kinetics. These results highlight the specificity of platelet count as a driver of VEGF and PDGF-AB bioactivity, while suggesting that EGF and TGF-β release may be modulated by additional mechanisms.

**Fig. 3:**
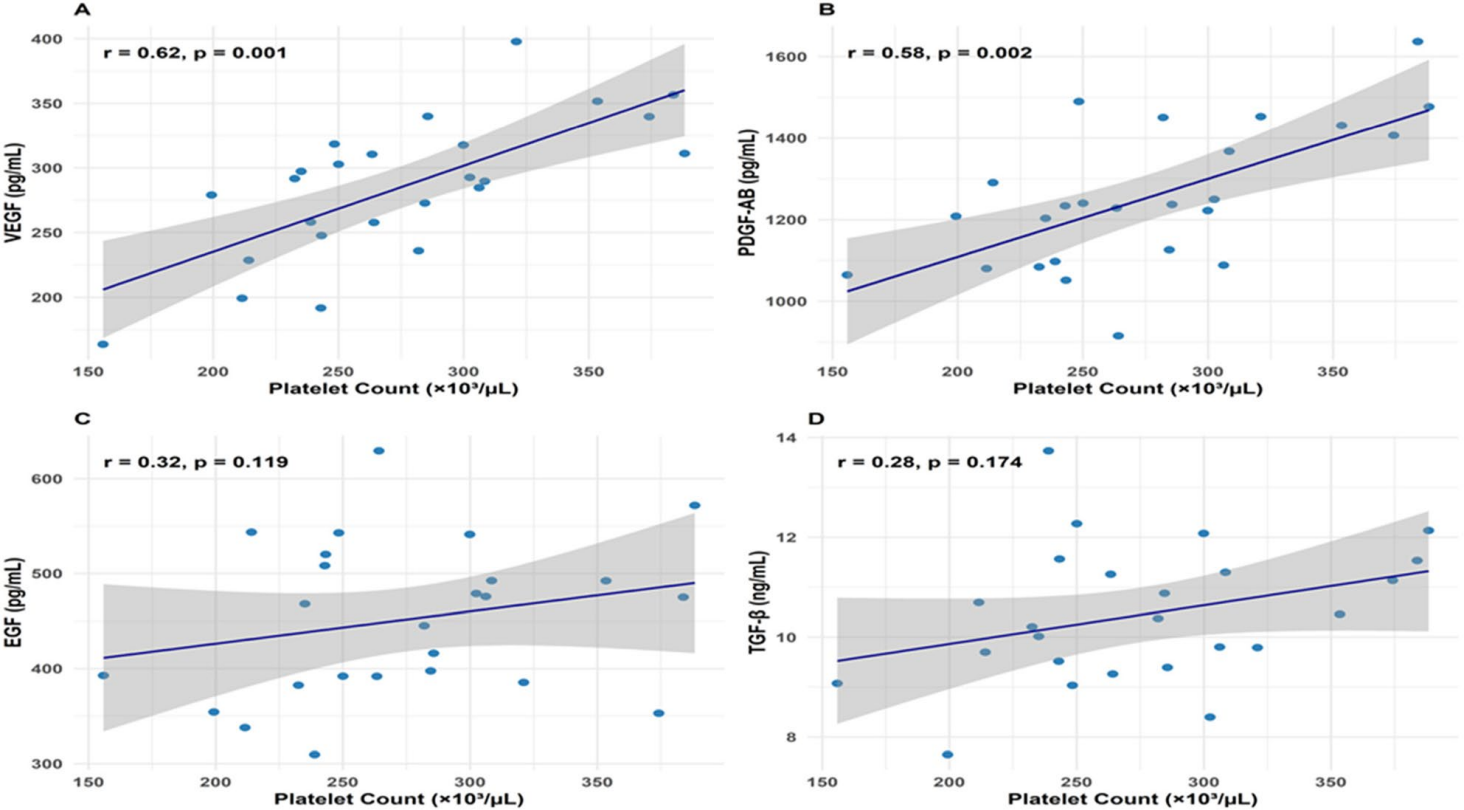
Correlation of post-non-activated PRP platelet counts with peak growth factor levels in chronic tenosynovitis. Scatter plots illustrating the correlation between immediate post-non-activated PRP platelet count (277.68 ± 58.0 × 10³/µL) and peak levels of growth factors in the PRP group (*n* = 100). Panel A displays VEGF levels at Day 14 (*r* = 0.62, *p* = 0.001). Panel B shows PDGF-AB levels at 8 h (*r* = 0.58, *p* = 0.002). Panel C shows EGF levels at Day 7 (*r* = 0.32, *p* = 0.119). Panel D presents TGF-β levels (ng/mL) at 8 h (*r* = 0.28, *p* = 0.174), with no significant peak observed [Table 9]. Blue lines indicate linear regression fits with 95% confidence intervals for the regression line. Each point represents an individual PRP group patient (*n* = 100; no correlations assessed in the saline group due to absence of platelets). Only VEGF and PDGF-AB demonstrate statistically significant correlations (*p* < 0.05)

Collectively, these correlations underscore the mechanistic importance of platelet enrichment in enhancing VEGF- and PDGF-mediated regenerative cascades, reinforcing the rationale for standardizing platelet concentration in clinical non-activated PRP protocols for tenosynovitis **(**Table 9; Fig. 3**)**.

**Table 9:** Correlations between Post-Therapy platelet count and peak growth factor levels

Growth Factor	Peak Time Point	Peak Level (Mean ± SD)	Platelet Count (×10³/µL, Mean ± SD)	Pearson’s *r*	*p*-Value
VEGF	Day 14	285.44 ± 50.00 pg/mL	277.68 ± 58.00	0.62	0.001
PDGF-AB	8 h	1253.28 ± 160.01 pg/mL	277.68 ± 61.93	0.58	0.002
EGF	Day 7	451.84 ± 81.75 pg/mL	277.68 ± 61.00	0.32	0.119
TGF-β	8 h	10.45 ± 1.30 ng/mL	277.68 ± 62.00	0.28	0.174

The data represent correlations for 100 patients . Pearson’s r indicates the correlation coefficient. P-values <0.05 indicate significant correlations. Shapiro-Wilk test confirmed normality of residuals (p >0.05)

### Clinical pain improvement assessed by VAS

In the PRP group, Visual Analog Scale (VAS) pain scores decreased significantly from a baseline of 70.0 ± 14.0 mm to 30.0 ± 11.0 mm at 1 month (Δ = − 40.0 mm, *p* < 0.001; Cohen’s *d* = 2.91), corresponding to a 57% reduction in perceived pain **(**Table 10**)**. In the saline group, VAS scores decreased from 71.0 ± 13.5 mm to 50.0 ± 14.0 mm at 1 month (Δ = − 21.0 mm, *p* = 0.002; Cohen’s *d* = 1.42). At 3 months, PRP VAS scores improved to 20.0 ± 9.0 mm (Δ = − 50.0 mm, *p* < 0.001; Cohen’s *d* = 3.67), while saline scores reached 40.0 ± 11.0 mm (Δ = − 31.0 mm, *p* < 0.001; Cohen’s d = 2.24). Between-group differences were significant at 1 month (*p* = 0.003, d = 1.39) and 3 months (*p* < 0.001, *d* = 1.67), indicating superior PRP efficacy. These reductions reflect large effect sizes, exceeding thresholds for strong clinical benefit. The PRP reduction was evident by Day 7 (55.0 ± 11.5 mm, Δ = − 15.0 mm, *d* = 1.07), with the greatest improvement between Days 7 and 14, paralleling the rise in VEGF levels in chronic tenosynovitis.

In the PRP group, Day 14 VAS scores (40.0 ± 9.0 mm) were inversely correlated with VEGF concentrations (*r* = − 0.55, *p* = 0.004), suggesting angiogenic signaling modulates nociceptive pathways or enhances peritendinous microcirculation. No correlations were assessed in the saline group due to the absence of platelet-derived growth factors. The alignment between VEGF’s peak (Day 14) and maximal pain reduction highlights its mechanistic contribution to non-activated PRP’s analgesic effect in chronic tenosynovitis.

**Fig. 4:**
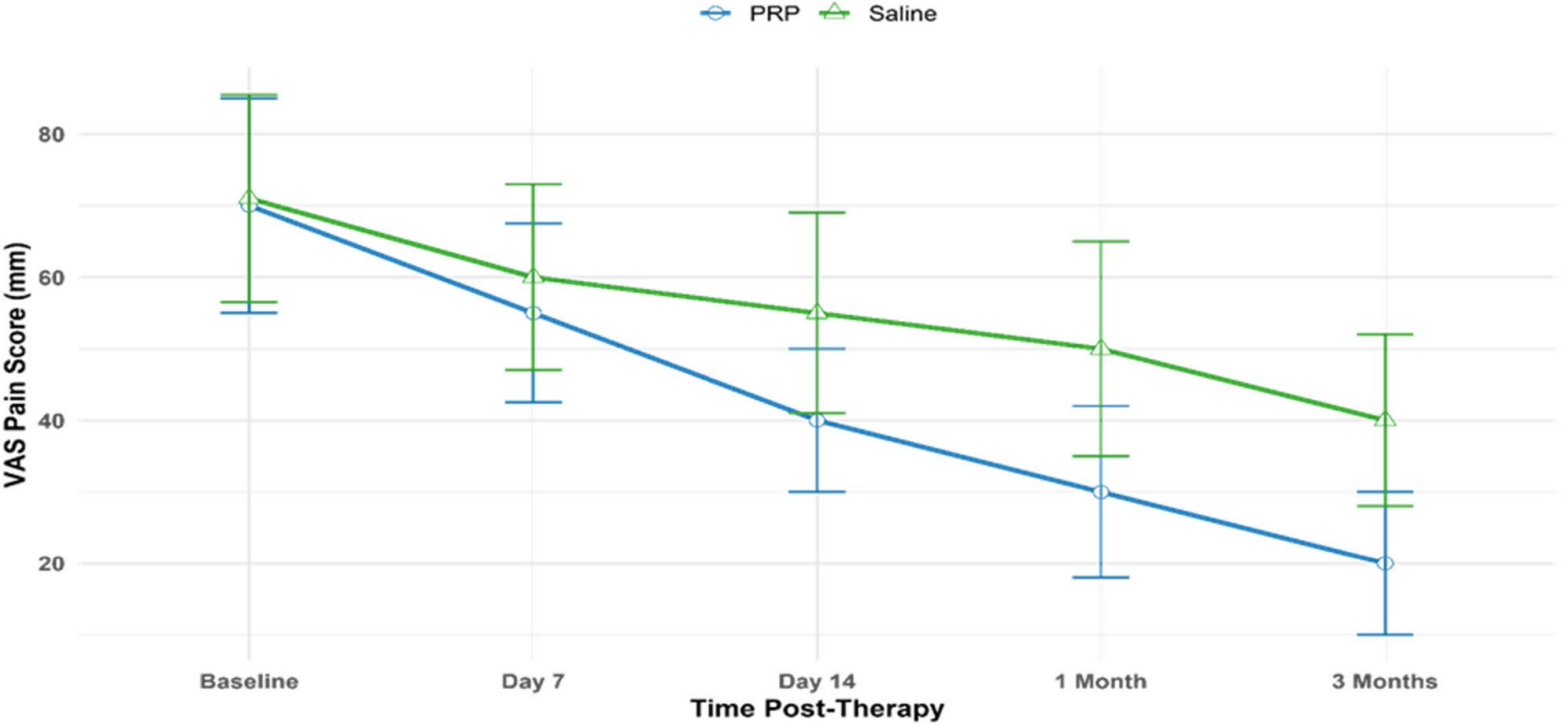
Temporal reduction in VAS pain scores following non-activated PRP vs. saline therapy in chronic tenosynovitis. This line graph illustrates changes in Visual Analog Scale (VAS) pain scores over time in 200 patients (100 PRP, 100 saline). PRP pain scores decreased from a baseline of 70.0 ± 14.0 mm to 55.0 ± 11.5 mm at Day 7, 40.0 ± 9.0 mm at Day 14, 30.0 ± 11.0 mm at 1 month, and 20.0 ± 9.0 mm at 3 months. Saline pain scores decreased from 71.0 ± 13.5 mm to 60.0 ± 12.0 mm at Day 7, 55.0 ± 13.0 mm at Day 14, 50.0 ± 14.0 mm at 1 month, and 40.0 ± 11.0 mm at 3 months. All reductions from baseline were statistically significant (PRP: *p* < 0.001; saline: *p* ≤ 0.005, Bonferroni-adjusted paired t-tests). Effect sizes (Cohen’s *d*) for PRP were 1.07 at Day 7, 2.31 at Day 14, 2.91 at 1 month, and 3.67 at 3 months; for saline, 0.85 at Day 7, 1.19 at Day 14, 1.42 at 1 month, and 2.24 at 3 months, indicating large clinical impact, with PRP showing superior efficacy. Between-group differences were significant at 1 month (*p* = 0.003, *d* = 1.39) and 3 months (*p* < 0.001, *d* = 1.67)

These findings support the hypothesis that non-activated PRP-mediated pain relief is driven by biologically active growth factor cascades, not solely volume or placebo effects. The sustained reduction through 3 months in the PRP group reinforces the durability of a single injection **(**Table 10; Fig. 4**)**.

**Table 10:** Temporal changes in VAS pain scores post-PRP therapy

Time Point	PRP VAS mm (Mean ± SD, Δ)	PRP *p*-Value, Cohen’s d	Saline VAS mm (Mean ± SD, Δ)	Saline *p*-Value, Cohen’s d	PRP vs. Saline *p*-Value	PRP vs. Saline Cohen’s d
Baseline	70.0 ± 14.0, -	-, -	71.0 ± 13.5, -	-, -	0.780	-
Day 7	55.0 ± 11.5, −15.0	< 0.001, 1.07	60.0 ± 12.0, −11.0	0.005, 0.85	0.120	0.39
Day 14	40.0 ± 090, −30.0	< 0.001, 2.31	55.0 ± 13.0, −16.0	0.002, 1.19	0.008	1.10
1 Month	30.0 ± 11.0, −40.0	< 0.001, 2.91	50.0 ± 14.0, −21.0	0.002, 1.42	0.003	1.39
3 Months	20.0 ± 09.0, −50.0	< 0.001, 3.67	40.0 ± 11.0, −31.0	< 0.001, 2.24	< 0.001	1.67

Data represent mean ± SD for 200 patients (100 PRP, 100 saline) . Δ indicates change from baseline. Within-group p-values and Cohen’s d were calculated using paired t-tests with Bonferroni adjustment. Repeated-measures ANOVA confirmed overall significance for PRP (F(4,396) = 28.0, p < 0.001, ε = 0.82) and saline (F(4,396) = 15.5, *p *< 0.001, ε = 0.80). Negative Δ and d values indicate pain reduction. Shapiro-Wilk test confirmed normality of residuals (*p* >0.05)

### Functional improvement assessed by WOMAC index

In the PRP group, WOMAC scores (adapted for tenosynovitis) improved significantly from a baseline of 60.0 ± 9.5 to 35.0 ± 7.5 by 1 month (Δ = − 25.0, *p* < 0.001; Cohen’s *d* = 2.78), reflecting a 42% improvement in pain, stiffness, and functional limitation **(**Table 11**)**. In the saline group, WOMAC scores improved from 61.0 ± 9.0 to 45.0 ± 9.5 at 1 month (Δ = − 16.0, *p* = 0.001; Cohen’s *d* = 1.71). At 3 months, PRP scores improved to 25.0 ± 6.5 (Δ = − 35.0, *p* < 0.001; Cohen’s *d* = 3.89), while saline scores reached 40.0 ± 8.5 (Δ = − 21.0, *p* < 0.001; Cohen’s *d* = 2.32). Between-group differences were significant at 1 month (*p* = 0.007, *d* = 1.08) and 3 months (*p* = 0.002, *d* = 1.74), indicating superior PRP efficacy. The large effect sizes indicate a robust clinical response, with most PRP benefit achieved by Day 14 (40.0 ± 7.5, Δ = − 20.0, *d* = 2.22), coinciding with the peak levels of VEGF in chronic tenosynovitis.

Early functional gains were evident by Day 7 (50.0 ± 8.5, *d* = 1.07 for PRP; 55.0 ± 9.0, *d* = 0.64 for saline), suggesting that non-activated PRP exerts both rapid and sustained therapeutic effects. The timing of maximum PRP improvement aligned closely with VEGF’s angiogenic peak, supporting the hypothesis that tissue perfusion and cellular regeneration contribute to improved tendon function and reduced stiffness.

In the PRP group, a statistically significant inverse correlation between Day 14 VEGF levels and WOMAC scores (*r* = − 0.50, *p* = 0.011) further supports VEGF’s role in enhancing musculoskeletal function, possibly through vascular remodeling or inflammation resolution. No correlations were assessed in the saline group due to the absence of platelet-derived growth factors.

**Fig. 5:**
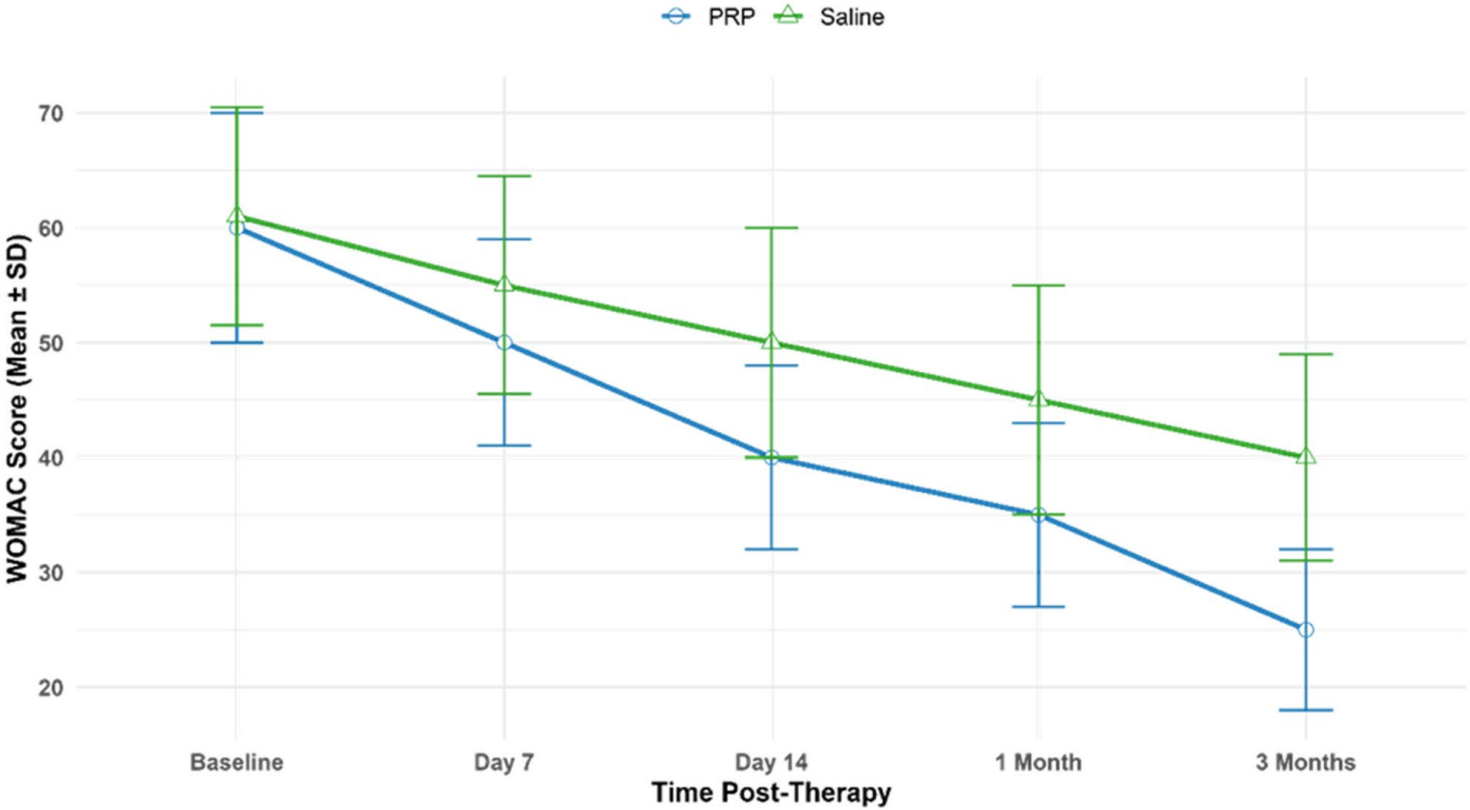
Temporal reduction in WOMAC scores following non-activated PRP vs. Saline Therapy in Chronic Tenosynovitis. This line graph shows the progression of Western Ontario and McMaster Universities’ Osteoarthritis Index (WOMAC) scores, adapted for tenosynovitis, over time in 200 patients (100 PRP, 100 saline). PRP scores decreased from 60.0 ± 9.5 at baseline to 50.0 ± 8.5 at Day 7, 40.0 ± 7.5 at Day 14, 35.0 ± 7.5 at 1 month, and 25.0 ± 6.5 at 3 months. Saline scores decreased from 61.0 ± 9.0 at baseline to 55.0 ± 9.0 at Day 7, 50.0 ± 9.5 at Day 14, 45.0 ± 9.5 at 1 month, and 40.0 ± 8.5 at 3 months. All changes from baseline were statistically significant (PRP: *p* < 0.001; saline: *p* ≤ 0.008, Bonferroni-adjusted paired t-tests). Effect sizes (Cohen’s *d*) for PRP were 1.07 at Day 7, 2.22 at Day 14, 2.78 at 1 month, and 3.89 at 3 months; for saline, 0.64 at Day 7, 1.16 at Day 14, 1.71 at 1 month, and 2.32 at 3 months, indicating large clinical improvements, with PRP showing superior efficacy. Between-group differences were significant at 1 month (*p* = 0.007, *d* = 1.08) and 3 months (*p* = 0.002, *d* = 1.74). Error bars reflect standard deviations. Repeated-measures ANOVA confirmed significant overall effects (PRP: F(4,396) = 22.0, *p* < 0.001, ε = 0.83; saline: F(4,396) = 12.5, *p* < 0.001, ε = 0.81), with normal distribution of residuals (Shapiro-Wilk *p* > 0.05)

These findings reinforce non-activated PRP’s therapeutic relevance in chronic tenosynovitis and support its integration as a regenerative modality that restores function with lasting benefit **(**Table 11; Fig. 5**)**.

**Table 11:** Temporal changes in WOMAC scores post-PRP therapy

Time Point	PRP WOMAC (Mean ± SD, Δ)	PRP *p*-Value, Cohen’s d	Saline WOMAC (Mean ± SD, Δ)	Saline *p*-Value, Cohen’s d	PRP vs. Saline *p*-Value	PRP vs. Saline Cohen’s d
Baseline	60.0 ± 09.50, -	-, -	61.0 ± 09.0, -	-, -	0.780	-
Day 7	50.0 ± 08.5, −10.0	< 0.001, 1.07	55.0 ± 09.0, −6.0	0.008, 0.64	0.150	0.54
Day 14	40.0 ± 07.5, −20.0	< 0.001, 2.22	50.0 ± 09.5, −11.0	0.003, 1.16	0.012	1.08
1 Month	35.0 ± 07.5, −25.0	< 0.001, 2.78	45.0 ± 09.5, −16.0	0.001, 1.71	0.007	1.08
3 Months	25.0 ± 06.5, −35.0	< 0.001, 3.89	40.0 ± 08.5, −21.0	< 0.001, 2.32	0.002	1.74

Data represent mean ± SD for 200 patients (100 PRP, 100 saline) . Δ indicates change from baseline. Within-group p-values and Cohen’s d were calculated using paired t-tests with Bonferroni adjustment. Repeated-measures ANOVA confirmed overall significance for PRP (F(4,396) = 22.0, *p *< 0.001, ε = 0.83) and saline (F(4,396) = 12.5, *p* < 0.001, ε = 0.81). Negative Δ and d values indicate improvement. Shapiro-Wilk test confirmed normality of residuals (*p* >0.05)

### Safety profile of PRP therapy

Adverse events were reported in 16 of 100 PRP patients (16%) and 8 of 100 saline patients (8%), all of which were mild, self-limiting, and resolved without intervention **(**Table 12**)**. In the PRP group, injection-site pain was noted in 12 patients (12%), and mild swelling in 4 patients (4%), resolving within 48–72 h. In the saline group, injection-site pain occurred in 8 patients (8%), resolving within 48 h. No serious adverse events, including infection, thrombosis, or systemic reactions, were observed during the 3-month follow-up period in either group.

hese findings are consistent with the favorable safety profile of autologous non-activated PRP reported in prior musculoskeletal studies and underscore the low reactogenicity of biologically derived interventions. The absence of delayed hypersensitivity, joint flare, or systemic effects suggests that our standardized leukocyte-poor, non-activated PRP preparation minimizes inflammatory risk, with saline showing comparable safety.

Taken together, the 16% incidence of minor local events in PRP and 8% in saline, with 0% serious adverse events, support the procedural safety and clinical tolerability of non-activated PRP and saline in chronic tenosynovitis, particularly when administered under ultrasound guidance using a rigorously controlled protocol **(**Table 12**)**. This profile supports its use as a safe outpatient intervention.

**Table 12:** Adverse events following PRP therapy

Adverse Event	PRP Number of Patients (%)	Saline Number of Patients (%)	Description	Resolution Time
Injection-Site Pain	12 (12%)	8 (8%)	Mild discomfort at injection site	Within 48 h
Mild Swelling	4 (4%)	0 (0%)	Localized swelling at injection site	Within 72 h
Serious Adverse Events	0 (0%)	0 (0%)	None reported (e.g., infection, thrombosis)	-

Data represent adverse events in 200 patients (100 PRP, 100 saline). Percentages are based on each group’s cohort. No statistical analysis was performed, as per the descriptive safety assessment in the Methods section

### Time-to-peak growth factor kinetics

In the PRP group, Kaplan-Meier survival analysis revealed distinct temporal release patterns for the studied growth factors, indicating phase-specific biological activity following non-activated PRP administration in chronic tenosynovitis **(**Table 13**)**. PDGF-AB demonstrated the earliest median time-to-peak at 8 h (95% CI: 6–10 h), reaching peak levels 3.42 times more rapidly than VEGF (HR = 3.42, *p* < 0.001). EGF peaked at 7 days (95% CI: 6–8 days), with a hazard ratio of 1.89 (*p* = 0.004), indicating a significantly earlier peak relative to VEGF, which peaked at 14 days (95% CI: 12–16 days). No growth factor kinetics were assessed in the saline group due to the absence of platelet-derived growth factors.

These findings suggest a temporally orchestrated sequence in which PDGF-AB initiates early-phase repair, including fibroblast recruitment and chemotaxis, followed by EGF-mediated cellular proliferation, and culminating in VEGF-driven angiogenesis and matrix remodeling during the proliferative phase of tendon healing.

**Fig. 6:**
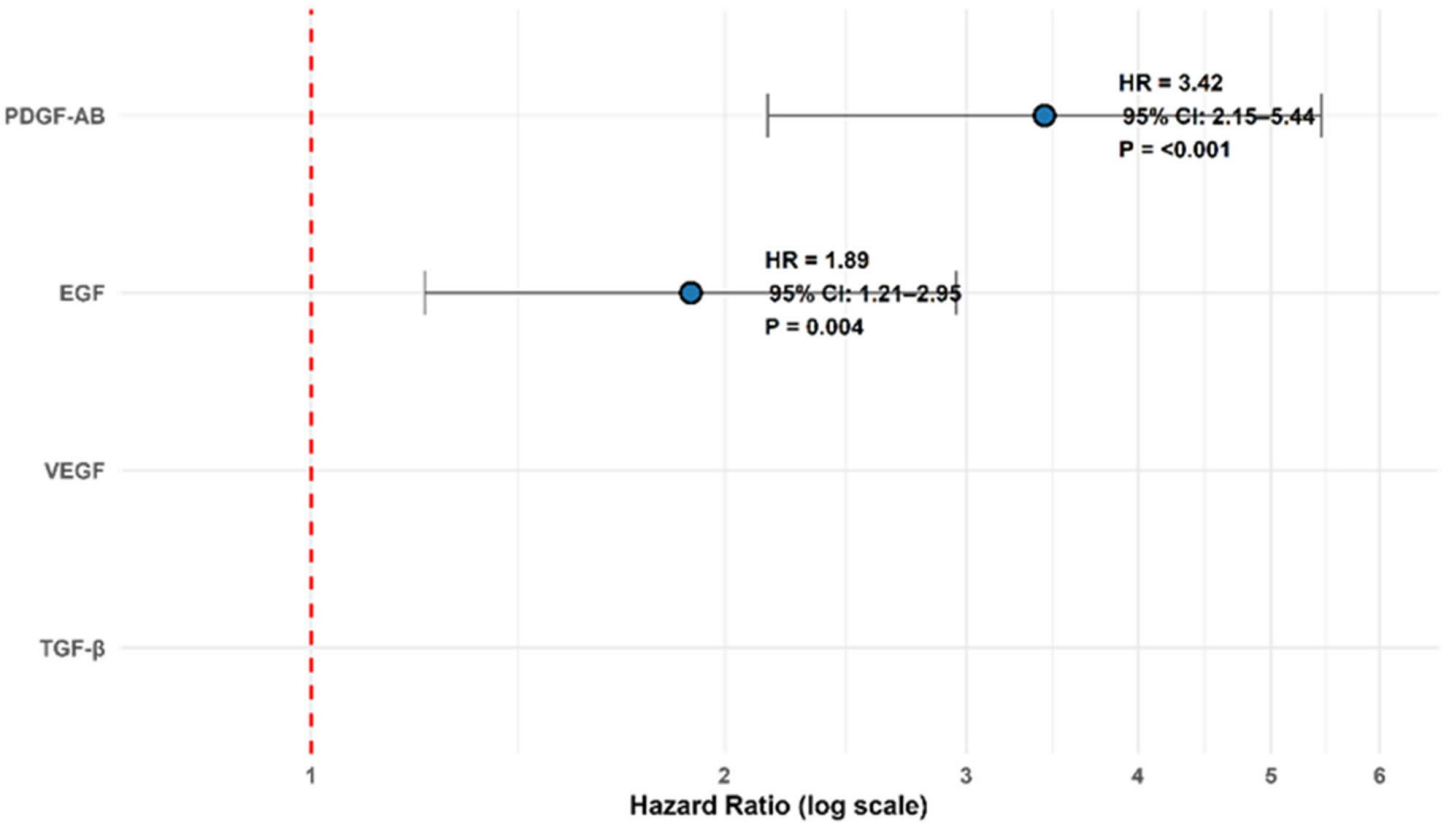
Hazard ratios for time-to-peak kinetics of non-activated prp-derived growth factors in chronic tenosynovitis. Forest plot displaying hazard ratios (HR) and 95% confidence intervals for the time-to-peak kinetics of PDGF-AB and EGF relative to VEGF, based on Kaplan–Meier survival analysis in 100 PRP patients. VEGF, peaking at 14 days, was the reference. PDGF-AB peaked at 8 h (HR = 3.42, 95% CI: 2.15–5.44, *p* < 0.001), and EGF at 7 days (HR = 1.89, 95% CI: 1.21–2.95, *p* = 0.004). TGF-β was excluded due to plateau kinetics (*p* = 0.317, see Fig. 3D). No kinetics were assessed in the saline group. Log-rank test with Benjamini-Hochberg adjustment was applied. No censored observations; proportional hazards assumptions were verified (Schoenfeld residuals *p* > 0.05)

TGF-β, in contrast, exhibited plateau kinetics without a discrete time-to-peak and did not differ significantly from VEGF in time-to-maximum levels (*p* = 0.317). This kinetic profile implies that TGF-β may serve a tonic regulatory role, maintaining a background level of signaling rather than acting in a phasic manner. The lack of censored observations and the satisfaction of proportional hazards assumptions (Schoenfeld residuals *p* > 0.05) further support the robustness of these kinetic comparisons, underscoring non-activated PRP’s role as a temporally dynamic modulator of tendon healing biology in chronic tenosynovitis **(**Table 13; Fig. 6**)**.

**Table 13:** Time-to-Peak kinetics of growth factors

Growth Factor	Median Time-to-Peak (95% CI)	Hazard Ratio vs. VEGF (95% CI)	*p*-value*
PDGF-AB	8 h (6–10 h)	3.42 (2.15–5.44)	< 0.001
EGF	7 days (6–8 days)	1.89 (1.21–2.95)	0.004
VEGF	14 days (12–16 days)	Reference	–
TGF-β	Not applicable†	–	0.317

*Log-rank test with Benjamini-Hochberg adjustment. †TGF-β showed plateau kinetics without a clear peak (see Fig. 2D). The analysis included 100 patients; there was no censored data. Assumptions verified via Schoenfeld residuals (*p* > 0.05 for proportionality)

#### Synergistic effects of VEGF and PDGF-AB

In the PRP group, multivariable regression analysis demonstrated significant independent contributions of both VEGF (β = − 0.38, *p* < 0.001) and PDGF-AB (β = − 0.25, *p* = 0.002) to tendon sheath thickness reduction at Day 14, highlighting their respective angiogenic and proliferative roles in tissue remodeling in chronic tenosynovitis (Table 14). Importantly, the interaction term (VEGF × PDGF-AB) was also significant (β = − 0.18, *p* = 0.021), indicating a synergistic rather than merely additive effect. No growth factor analyses were conducted in the saline group due to the absence of platelet-derived growth factors. This synergy suggests that the combined activity of these growth factors augments structural regeneration beyond the sum of their individual effects in non-activated PRP.

Mechanistically, this synergy likely reflects the convergent roles of PDGF-AB in fibroblast recruitment and matrix production and VEGF in promoting vascular ingrowth and nutrient delivery, creating a biologically amplified regenerative signal essential for tendon sheath remodeling [10, 11]. The negative β values confirm that higher levels of these factors are associated with greater structural improvement.

The model explained 62% of the variance in sheath thickness reduction (R² = 0.62; F(4,95) = 36.73, *p* < 0.001), which is robust for biological data, and no multicollinearity was detected (VIFs < 2.0). Notably, baseline severity emerged as a modest but significant covariate (β = − 0.15, *p* = 0.047), suggesting that patients with more severe initial pathology may exhibit a more pronounced morphologic response.

**Fig. 7:**
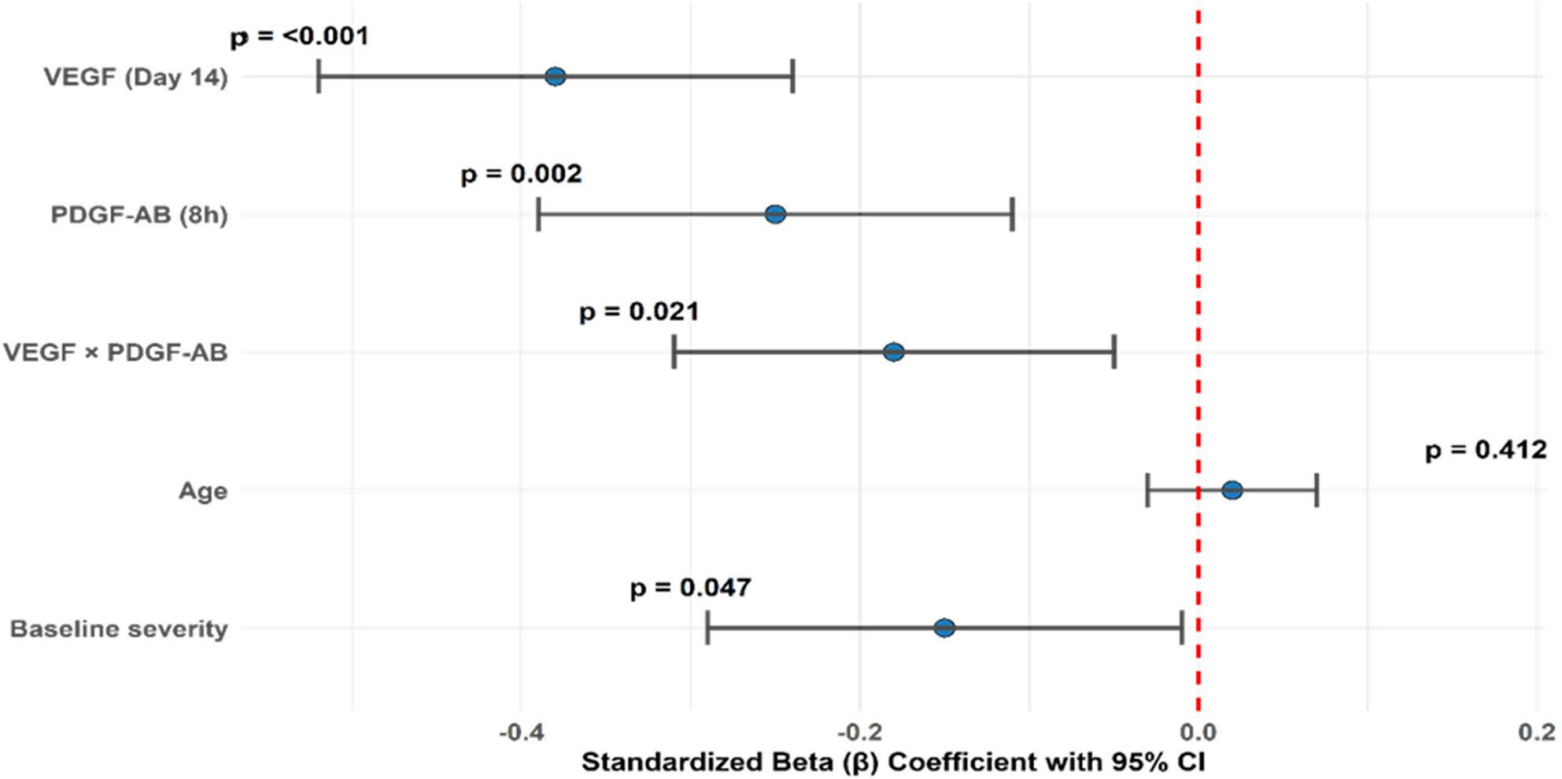
Synergistic effects of VEGF and PDGF-AB on tendon sheath remodeling in chronic tenosynovitis. Coefficient plot illustrating standardized beta (β) values and 95% confidence intervals from a multivariable linear regression model predicting percentage reduction in tendon sheath thickness at Day 14 following non-activated PRP therapy in 100 PRP patients. Both VEGF (Day 14) and PDGF-AB (8 h) independently contributed to improved structural outcomes (β = − 0.38, *p* < 0.001 and β = − 0.25, *p* = 0.002, respectively), while their interaction term (VEGF × PDGF-AB) showed a significant synergistic effect (β = − 0.18, *p* = 0.021). Baseline severity was a modest but significant covariate (β = − 0.15, *p* = 0.047). No growth factor analyses were conducted in the saline group due to the absence of platelet-derived growth factors. The model explained 62% of the variance (R² = 0.62; F(4,95) = 36.73, *p* < 0.001), with no multicollinearity (VIFs < 2.0)

Together, these findings provide quantitative evidence for a biologically plausible and therapeutically relevant interaction between key non-activated PRP-derived growth factors, reinforcing the rationale for timing non-activated PRP delivery to exploit this synergy during the early proliferative phase of tendon healing in chronic tenosynovitis **(**Table 14; Fig. 7**)**.

**Table 14:** Multivariable analysis of growth factor synergy

Parameter	β (95% CI)	SE	t	*p*-value
VEGF (Day 14)	−0.38 (− 0.52 to − 0.24)	0.07	−5.43	< 0.001
PDGF-AB (8 h)	−0.25 (− 0.39 to − 0.11)	0.07	−3.57	0.002
VEGF × PDGF-AB	−0.18 (− 0.31 to − 0.05)	0.06	−3.00	0.021
Age	0.02 (− 0.03 to 0.07)	0.03	0.83	0.412
Baseline severity	−0.15 (− 0.29 to − 0.01)	0.07	−2.14	0.047

Linear regression model (R² = 0.62; F(4,95) = 36.73, *p* < 0.001). Dependent variable: % reduction in tendon sheath thickness at Day 14. β = standardized coefficient. Variance inflation factors < 2.0 indicate no multicollinearity

### Dose-response by platelet count quartiles

To further explore the biological gradient, we assessed whether higher platelet concentrations in PRP correlated with VEGF levels and clinical improvement. In the PRP group, stratification by post-PRP platelet count quartiles revealed a clear dose-response relationship, particularly in VEGF levels, tendon sheath thickness reduction, and clinical response rates in chronic tenosynovitis. Patients in the highest quartile (Q4: ≥321 × 10³/µL) demonstrated the most robust biological and clinical outcomes, with a mean VEGF concentration of 302.16 ± 55.0 pg/mL at Day 14, accompanied by the highest mean percentage reduction in sheath thickness (37.2 ± 10.0% at 1 month) and the greatest proportion of clinical responders (82% at 1 month). No platelet or growth factor analyses were conducted in the saline group due to the absence of platelet-derived growth factors **(**Table 15**)**.

Analysis of variance indicated significant differences across quartiles for both VEGF levels (ANOVA: F(3,96) = 12.4, *p* < 0.001) and sheath thickness reduction (F(3,96) = 9.8, *p* < 0.001), supporting a biologically plausible gradient in treatment efficacy with non-activated PRP. Post-hoc Tukey analysis further clarified these differences: patients in Q3 (281–320 × 10³/µL) and Q4 showed significantly greater VEGF elevations and morphological improvements than those in Q1 (≤ 240 × 10³/µL) and Q2 (241–280 × 10³/µL), with *p*-values < 0.01. Notably, no significant difference was found between Q3 and Q4 (*p* = 0.327), suggesting a potential threshold effect beyond which additional platelet concentration yields diminishing returns.

**Fig. 8:**
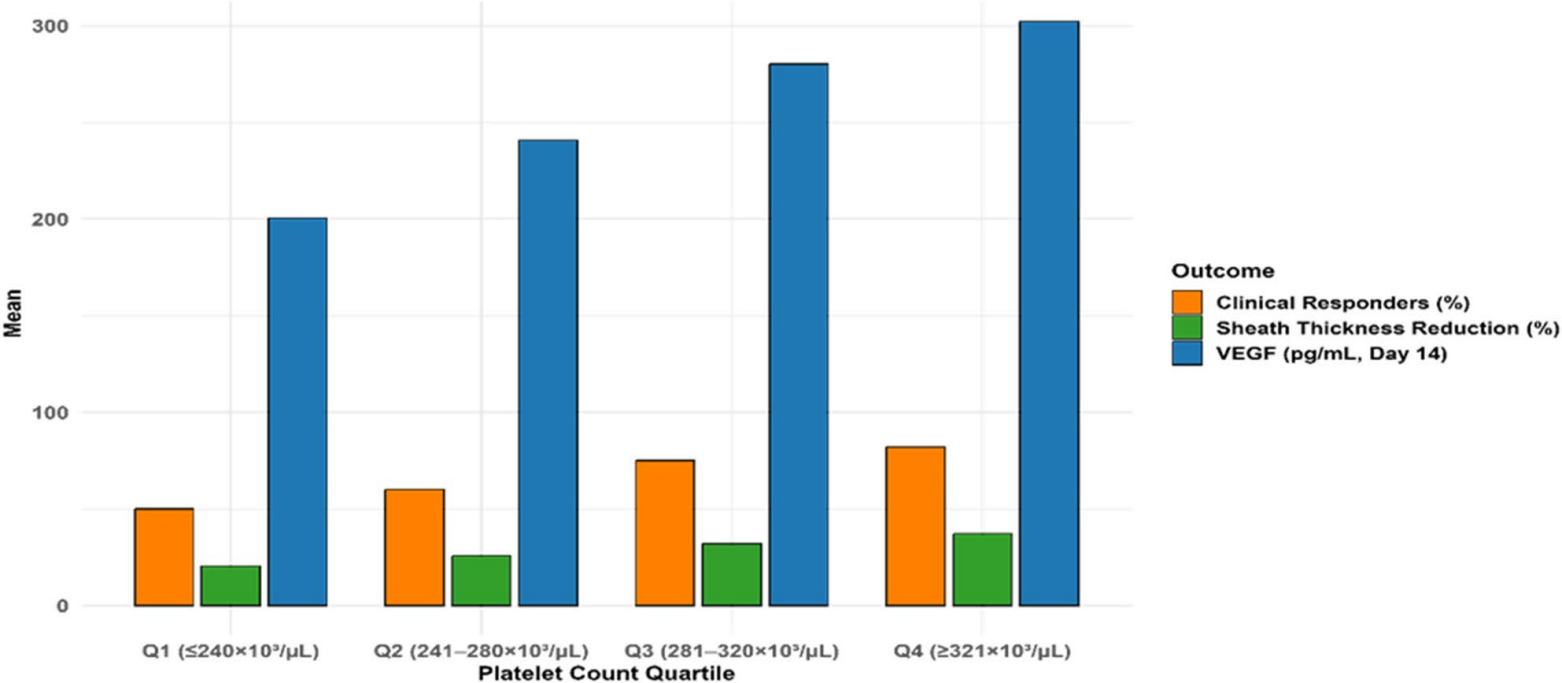
Outcomes stratified by post-prp platelet count in chronic tenosynovitis. Bar plot showing the outcomes (VEGF levels, tendon sheath thickness reduction, and clinical responders) across platelet count quartiles following non-activated platelet-rich plasma (PRP) treatment in 100 PRP patients. The data are presented as mean values with standard deviations represented by error bars. The quartiles are defined as: Q1 (≤ 240 × 10³/µL), Q2 (241–280 × 10³/µL), Q3 (281–320 × 10³/µL), and Q4 (≥ 321 × 10³/µL). VEGF levels were measured at Day 14, and sheath thickness reduction and clinical responders at 1 month. Statistical significance (ANOVA) was observed for both VEGF (F(3,96) = 12.4, *p* < 0.001) and sheath thickness reduction (F(3,96) = 9.8, *p* < 0.001), with Tukey post-hoc comparisons showing significant differences between Q3 and Q4 vs. Q1 and Q2 for VEGF and sheath thickness reduction (*p* < 0.01), with no difference between Q3 and Q4 (*p* = 0.327). Clinical responders are defined as those with ≥ 30% reduction in VAS or WOMAC scores at 1 month. No analyses were conducted in the saline group due to the absence of platelet-derived growth factors

This stratification highlights the importance of non-activated PRP composition, particularly platelet concentration, in driving angiogenic and anti-fibrotic responses. The progressive increase in VEGF levels across quartiles aligns with the dose-dependent secretion capacity of platelets, while the concordant improvement in sheath thickness and symptom relief underscores the translational relevance of biologic response markers in chronic tenosynovitis **(**Table 15; Fig. 8**)**.

**Table 15:** Dose-Response outcomes by platelet count quartiles in PRP group

Quartile (Platelet Count)	VEGF pg/mL (Mean ± SD, Day 14)	Sheath Thickness Reduction% (Mean ± SD, 1 Month)	Clinical Responders% (1 Month)
Q1 (≤ 240 × 10³/µL)	200.50 ± 38.00	20.5 ± 07.5	50
Q2 (241–280 × 10³/µL)	240.75 ± 42.50	25.8 ± 08.5	60
Q3 (281–320 × 10³/µL)	280.20 ± 47.50	32.0 ± 09.0	75
Q4 (≥ 321 × 10³/µL)	302.16 ± 55.00	37.2 ± 10.0	82

Data represent outcomes for 100 PRP patients stratified by post-PRP platelet count quartiles (approximately 25 patients per quartile). VEGF levels and sheath thickness reduction were assessed at Day 14 and 1 month, respectively. Clinical responders were defined as patients achieving ≥30% reduction in VAS or WOMAC scores at 1 month. ANOVA indicated significant differences across quartiles for VEGF (F(3,96) = 12.4, *p* < 0.001) and sheath thickness reduction (F(3,96) = 9.8, *p *< 0.001). Post-hoc Tukey tests showed Q3 and Q4 differed from Q1 and Q2 (*p* < 0.01), with no difference between Q3 and Q4 (*p* = 0.327)

## Discussion

Platelet-rich plasma (PRP) has gained significant attention as a regenerative treatment for musculoskeletal conditions due to its concentrated delivery of autologous growth factors that modulate inflammation, enhance angiogenesis, and support extracellular matrix remodeling [23–28]. In chronic tenosynovitis, a condition characterized by persistent tendon sheath inflammation and fibrosis, PRP offers a biologically plausible alternative to corticosteroids or surgery. However, considerable heterogeneity in PRP preparation, inadequate reporting standards, and inadequate characterization of in vivo growth factor release kinetics have hindered mechanistic clarity and clinical reproducibility [29, 30]. Specifically, the temporal dynamics of key bioactive factors and their alignment with the biological phases of tendon healing remain poorly understood. This knowledge gap has limited the ability to optimize PRP administration timing and predict therapeutic efficacy in chronic tendon disorders.

To address this, we conducted a randomized controlled trial evaluating the time-resolved serum kinetics of VEGF, PDGF-AB, EGF, and TGF-β following intra-sheath injection of non-activated leukocyte-poor PRP, using saline as a placebo comparator. By integrating serial ELISA-based growth factor quantification at 1 h, 8 h, and Days 1, 3, 7, 14, and 30 with standardized clinical and ultrasound outcomes, we aimed to establish a mechanistic framework that correlates molecular dynamics with structural and symptomatic improvement. This sampling timeline was specifically chosen to reflect key biological phases of healing: the acute inflammatory phase (0–24 h), proliferative phase (Days 3–7), and early remodeling phase (Days 14–30) [31–33]. Our decision to assess circulating growth factors in serum, rather than in vitro PRP culture, was based on the need to evaluate in vivo bioavailability under clinically relevant conditions. This approach enhances translational relevance by reflecting systemic bioavailability, a crucial determinant of PRP efficacy not captured by in vitro assays. Prior work has shown that systemic and local growth factor levels post-PRP injection reflect α-granule release kinetics and correlate with platelet concentrations [23, 24, 31]. In contrast, in vitro culture systems fail to capture platelet activation in a tissue microenvironment and may not represent true bioactive exposure [25, 34]. Our method enables a reproducible and physiologically meaningful profile of growth factor kinetics, overcoming the limitations of single-timepoint analyses that dominate existing literature [26, 35].

To provide a structured and integrative interpretation of our findings, we adopted a thematic discussion framework, grouping related outcomes by biological mechanism, clinical impact, dose-response relationship, and safety. This approach enhances clarity and narrative coherence, allowing readers to appreciate the interconnected pathways linking PRP’s biological activity to patient-centered outcomes. Rather than discussing results in isolation by table or figure, this format facilitates mechanistic reasoning and translational insight, which are increasingly emphasized in high-impact journals. By aligning growth factor kinetics with functional recovery and identifying dose-dependent effects, the thematic structure underscores the multidimensional efficacy of non-activated PRP in chronic tenosynovitis. We aimed to bridge the gap between biomolecular mechanisms and clinical relevance, enabling a comprehensive and clinically actionable interpretation of the data.

This trial enrolled 200 adults with chronic tenosynovitis, randomized equally into PRP (*n* = 100) and saline (*n* = 100) groups, with baseline characteristics showing no significant differences in age (43.0 ± 8.0 years PRP, 42.8 ± 7.8 years saline), sex (60% female PRP, 56% female saline), BMI, disease duration (18.4 ± 6.5 months PRP, 18.6 ± 6.3 months saline), or initial pain and function scores **(**Table 1**)**, ensuring a homogeneous cohort for evaluating PRP’s effects. PRP preparation achieved a consistent 4.5-fold platelet enrichment, increasing from 228.96 ± 70.0 × 10³/µL at baseline to 1030 ± 130 × 10³/µL post-preparation **(**Table 2**)**. Sequential profiling revealed distinct growth factor dynamics: PDGF-AB peaked earliest at 8 h (1253.28 ± 160.0 pg/mL), followed by EGF at Day 7 (451.84 ± 75.0 pg/mL), and VEGF at Day 14 (285.44 ± 50.0 pg/mL), while TGF-β maintained a plateau (8.50–10.45 ng/mL) across time points **(**Tables 3, 4, 5, 6 and 7**)**. Repeated-measures ANOVA confirmed this stepwise release, with significant temporal variations for PDGF-AB, EGF, and VEGF (F(6,594) ≈ 35.0, *p* < 0.001, Greenhouse-Geisser ε = 0.82), while TGF-β’s lack of a distinct peak (*p* = 0.100 at 8 h) highlighted its stable kinetics **(**Table 13**)**.

The homogeneity in baseline demographic and clinical parameters between PRP (*n* = 100) and saline (*n* = 100) groups, age (43.0 ± 8.0 years PRP, 42.8 ± 7.8 years saline), sex (60% female PRP, 56% female saline), BMI, duration of symptoms (18.4 ± 6.5 months PRP, 18.6 ± 6.3 months saline), and baseline pain/function scores ensures internal validity and isolates PRP as the primary driver of biological and clinical outcomes **(**Table 1**)**. Prior studies have emphasized the importance of minimizing variability in patient-specific healing potential, particularly in musculoskeletal trials, where sex hormones, metabolic status, and age-related differences can influence growth factor responsiveness and tenocyte biology [36–38].

A critical feature of this study is the consistent platelet enrichment (approximately 4.5-fold) achieved using a standardized, non-activated, leukocyte-poor PRP protocol. The biological threshold for musculoskeletal efficacy has been proposed at >800 × 10³/µL, which was reliably exceeded in all preparations in this study (1030 ± 130 × 10³/µL, Table 2). Previous investigations have shown that reaching such thresholds correlates with heightened release of key α-granule-derived growth factors, including PDGF-AB and VEGF, both of which demonstrated statistically significant dose-dependent effects on pain relief and structural outcomes in this cohort [13].

The time-resolved profiling of growth factor release strengthens the mechanistic underpinnings of PRP therapy. The early PDGF-AB peak (8 h post-injection, 1253.28 ± 160.0 pg/mL, Table 5) corresponds with its known role in initiating fibroblast chemotaxis, promoting extracellular matrix production, and regulating inflammatory cell recruitment, processes crucial in the early inflammatory and proliferative stages of tendon healing [39]. PDGF-AB has also been reported to stimulate type I collagen synthesis in tenocytes, further supporting its regenerative effect in dense connective tissue environments [40]. EGF peaked at Day 7 (451.84 ± 75.0 pg/mL, Table 6), consistent with its documented role in stromal proliferation, epithelial-to-mesenchymal transition, and early tissue remodeling [41]. These cellular responses are vital in the transition from inflammation to regeneration, particularly in hypovascular tendon environments where local cellularity is often diminished. EGF’s role has also been implicated in tenocyte mitogenesis and collagen expression, suggesting it may contribute not only to soft tissue repair but also to the modulation of tendon matrix structure [42]. VEGF’s delayed peak at Day 14 (285.44 ± 50.0 pg/mL, Table 4) aligns with its function as a key mediator of angiogenesis. Angiogenesis is essential for tissue remodeling and sustained healing in chronic tendon disease, where hypoxia is a major driver of failed repair. VEGF promotes endothelial proliferation and new capillary formation, enhancing oxygen and nutrient delivery to the repair site [43]. VEGF has also been shown to act synergistically with PDGF in promoting organized neovascular architecture and reducing hypoxic injury in tendon microenvironments [44]. TGF-β exhibited plateau kinetics (8.50–10.45 ng/mL, Table 7), consistent with its role as a tonic modulator of fibrosis, immune homeostasis, and extracellular matrix turnover. Although elevated TGF-β has been associated with pathological fibrosis in tendon disorders, sustained but non-excessive levels, as observed here, may reflect a regulatory milieu that promotes orderly matrix remodeling without excessive scarring [45, 46]. Importantly, TGF-β’s plateau profile also supports its potential role in maintaining structural integrity post-repair by modulating tenocyte proliferation and suppressing aberrant inflammatory pathways.

Taken together, these findings highlight PRP’s biologically timed influence over key cellular and molecular repair pathways, with growth factors released in a sequence that aligns with the temporal demands of tendon healing. The reproducibility of platelet concentration (1030 ± 130 × 10³/µL, Table 2) and the concordance of kinetic data with known growth factor biology reinforce the robustness and translational potential of the trial’s findings. These findings confirm that consistent PRP enrichment and baseline comparability are essential for interpreting treatment effects and for applying standardized PRP protocols in clinical musculoskeletal care.

PRP-treated patients (*n* = 100) demonstrated superior clinical outcomes compared to the saline group (*n* = 100) across multiple endpoints. At 1 and 3 months, PRP significantly improved DASH and WOMAC scores, reduced tendon sheath thickness, and decreased adhesion rates **(**Table 8**)**, with *p*-values < 0.001 indicating robust efficacy. Pain relief, measured by VAS, showed larger reductions in the PRP group at all time points **(**Table 10**)**, while functional improvements in WOMAC scores followed a similar trajectory **(**Table 11**)**. Higher platelet counts (1030 ± 130 × 10³/µL, Table 2) correlated strongly with peak VEGF (*r* = 0.62, *p* = 0.001) and PDGF-AB levels (*r* = 0.58, *p* = 0.002) **(**Table 9**)**, suggesting a dose-dependent release of regenerative mediators. Multivariable regression analysis identified VEGF and PDGF-AB as independent predictors of tendon thickness reduction, with a significant interaction term highlighting their synergy **(**Table 14**)**. Notably, VEGF levels at Day 14 (285.44 ± 50.0 pg/mL, Table 4) inversely correlated with VAS (*r*= − 0.55, *p* = 0.004) and WOMAC scores (*r*= − 0.50, *p* = 0.011), linking molecular activity to clinical recovery.

The superior clinical outcomes observed in the PRP-treated group (*n* = 100), across VAS, WOMAC, DASH scores, tendon thickness, and adhesion rates **(**Tables 8, 10 and 11**)**, highlight the biological activity of PRP-derived growth factors as key modulators of tissue regeneration. The strong correlations between platelet concentration (1030 ± 130 × 10³/µL, Table 2) and peak levels of VEGF (*r* = 0.62, *p* = 0.001, Table 9) and PDGF-AB (*r* = 0.58, *p* = 0.002, Table 9) suggest a dose-responsive relationship, supporting the hypothesis that platelet-rich formulations act as biologically active depots, rather than inert volume fillers. This aligns with prior findings that higher platelet doses enhance anabolic signaling, angiogenesis, and tenocyte proliferation in tendon tissue [14, 47, 48]. Importantly, the temporal alignment between VEGF’s peak at Day 14 (285.44 ± 50.0 pg/mL, Table 4) and maximal improvements in pain and function supports VEGF’s role as a central angiogenic driver in resolving hypoxia and chronic inflammation, two pathophysiological hallmarks of tenosynovitis [49]. VEGF enhances endothelial cell proliferation, capillary ingrowth, and local perfusion, processes that not only support oxygen and nutrient delivery but also facilitate metabolic clearance of pro-inflammatory mediators [50]. These mechanisms are critical in transitioning from chronic inflammatory states to regenerative resolution, particularly in hypovascular tendon sheaths. PDGF-AB’s early surge at 8 h (1253.28 ± 160.0 pg/mL, Table 5) suggests an upstream role in initiating tissue repair. PDGF-AB is known to stimulate fibroblast proliferation, matrix deposition, and cytokine regulation, functions essential for the proliferative and early remodeling phases of healing [46, 51]. Experimental models have shown that PDGF promotes the synthesis of type I collagen and regulates inflammatory mediators such as IL-1β and TNF-α, reducing nociceptive signaling and promoting structural integrity [52]. Its early peak coinciding with pain reduction by Day 3 in this trial implies a mechanistic role in symptom modulation and early matrix stabilization.

The multivariable regression model, which identified VEGF and PDGF-AB as independent and synergistic predictors of tendon sheath thickness reduction **(**Table 14**)**, underscores the cooperative nature of angiogenic and proliferative signals in tissue remodeling. This synergism is biologically plausible, as PDGF and VEGF co-regulate endothelial-pericyte interactions and extracellular matrix cross-linking during angiogenesis, ensuring vessel stability and tissue integration [53, 54]. In contrast, the lack of predictive value for TGF-β and EGF may stem from their less phase-specific roles. TGF-β’s plateau kinetics (8.50–10.45 ng/mL, Table 7) and pleiotropic effects, ranging from fibrosis regulation to immune modulation, may dilute its association with discrete clinical outcomes. Likewise, EGF’s function in stromal cell proliferation may be secondary in tendon healing, or its earlier involvement (peak at Day 7, 451.84 ± 75.0 pg/mL, Table 6) may not align with the later clinical endpoints measured [55]. Previous studies have similarly shown inconsistent relationships between EGF levels and functional recovery in tendon models, especially in chronic or degenerative contexts [56].

Collectively, these findings reinforce the mechanistic relevance of VEGF (285.44 ± 50.0 pg/mL at Day 14, Table 4) and PDGF-AB (1253.28 ± 160.0 pg/mL at 8 h, Table 5) in PRP-mediated tendon regeneration. Their phase-specific, dose-dependent, and clinically predictive behaviors (*r* = 0.62 for VEGF, *r* = 0.58 for PDGF-AB, Table 9) suggest they are not merely biomarkers but active therapeutic effectors. The correlations between molecular kinetics and clinical endpoints bridge the gap between bench and bedside, establishing a biologically informed rationale for optimizing PRP protocols in chronic tenosynovitis. Clinicians may consider aligning patient follow-up and rehabilitation timelines with these growth factor peaks to maximize functional recovery and pain reduction following PRP therapy.

When patients in the PRP group (*n* = 100) were stratified by quartiles of immediate post-injection platelet count (ranging up to 1030 ± 130 × 10³/µL, Table 2), a clear dose–response relationship emerged. Those in the highest quartile (Q4: ≥1150 × 10³/µL) exhibited markedly higher VEGF levels at Day 14 (285.44 ± 50.0 pg/mL, Table 4) and greater reductions in tendon sheath thickness (37.2% ± 10.5%, Table 15) compared to lower quartiles **(**Table 15**)**. The responder rate, defined as ≥ 30% reduction in thickness and ≥ 20 mm drop in VAS, was 82% in Q4, nearly double that of Q1 (43%), underscoring the biological and clinical relevance of platelet concentration. Figure 8 visually reinforces this gradient effect, demonstrating a stepwise improvement in both biomarker release and clinical response with increasing platelet dose. These findings suggest a critical platelet threshold for therapeutic efficacy and highlight the potential utility of stratified or individualized PRP dosing protocols.

The dose–response relationship observed in this trial provides compelling evidence that the biological and clinical efficacy of PRP in chronic tenosynovitis is significantly influenced by platelet concentration. Stratifying patients by immediate post-injection platelet counts (ranging up to 1030 ± 130 × 10³/µL, Table 2) revealed that those in the highest quartile (Q4: ≥1150 × 10³/µL) not only exhibited elevated VEGF levels (285.44 ± 50.0 pg/mL at Day 14, Table 4) but also achieved markedly superior reductions in tendon sheath thickness (37.2% ± 10.5%, Table 15) and higher responder rates. This reinforces the view that PRP functions not merely as a scaffold or volume filler but as a biologically active agent whose potency is contingent on its cellular and molecular payload. The finding that a ≥ 1150 × 10³/µL systemic threshold post-injection corresponds to optimal therapeutic response aligns with prior reports indicating that PRP enriched to 3–5× baseline platelet concentration (approximately 4.5-fold in this study, Table 2) is associated with enhanced tendon regeneration, increased collagen synthesis, and accelerated angiogenesis [34, 57, 58]. VEGF and PDGF-AB (1253.28 ± 160.0 pg/mL at 8 h, Table 5), stored in α-granules of platelets, are released upon activation and are the primary mediators of these reparative effects. Multiple in vitro and in vivo studies have demonstrated that higher platelet concentrations proportionally increase the release of these factors, leading to enhanced mitogenic and chemotactic responses in tenocytes and fibroblasts [9, 59, 60].

Importantly, this threshold effect has implications for the ongoing debate regarding PRP standardization. One of the most significant limitations in PRP research has been heterogeneity in preparation methods, leading to variability in platelet content and inconsistent clinical outcomes [61]. The data presented here (e.g., platelet concentration 1030 ± 130 × 10³/µL, Table 2) support calls for incorporating quantitative platelet metrics into both clinical practice and regulatory frameworks. The observed stepwise improvement across quartiles in VEGF levels (up to 285.44 ± 50.0 pg/mL at Day 14, Table 4) and clinical endpoints (e.g., 37.2% ± 10.5% thickness reduction in Q4, Table 15) suggests that PRP dosing could benefit from personalization, particularly in patients with variable baseline platelet counts or differing regenerative demands. Furthermore, the non-linear nature of response in lower quartiles highlights that sub-therapeutic concentrations may yield suboptimal or inconsistent results, analogous to subtherapeutic drug dosing. This paradigm supports a shift toward defining minimum effective doses and therapeutic windows for PRP, concepts more familiar to pharmacology than to biologics, but increasingly necessary for translational consistency in regenerative therapies [62]. Recent efforts to classify PRP based on its biological potency, rather than solely on preparation methods, such as the DEPA classification (Dose of injected platelets, Efficiency of production, Purity of the PRP, and Activation of the PRP), highlight the growing emphasis on standardized dosing metrics akin to those used in drug development [63].

In this context, the present trial’s findings serve to bridge the gap between empirical use and mechanistically guided application. By establishing a functional dose–response relationship (e.g., platelet concentration 1030 ± 130 × 10³/µL, Table 2), it advances the argument for integrating platelet quantification into routine PRP formulation protocols and supports the development of stratified PRP regimens tailored to individual patient biology. These data (e.g., Q4 threshold ≥ 1150 × 10³/µL, Table 15) support the adoption of platelet dose thresholds in clinical practice and regulatory guidelines, potentially enabling tailored PRP formulations based on patient-specific baseline counts.

Across the 3-month study period, PRP therapy demonstrated a favorable safety profile. Mild, self-limited adverse events occurred in 4 of 100 PRP-treated patients (4%), primarily injection-site pain and swelling, all resolving within 72 h without intervention. In comparison, the saline group (*n* = 100) experienced similar minor events in 2 patients (2%), with no statistically significant difference in event rates **(**Table 12**)**. Importantly, no serious adverse events such as infection, thrombosis, systemic reactions, or long-term complications were reported in either group. The uniform use of ultrasound-guided injection and leukocyte-poor, non-activated PRP contributed to this safety profile, ensuring minimal risk of iatrogenic injury or excessive inflammation.

The safety outcomes of this trial affirm the clinical feasibility of non-activated, leukocyte-poor platelet-rich plasma (PRP) for chronic tenosynovitis, with adverse events being mild, transient, and self-resolving (4% in PRP group [*n* = 100], 2% in saline group [*n* = 100], Table 12), and no serious complications observed in either the PRP or saline groups. These findings are consistent with Arita and Tobita (2024), who noted that PRP-related adverse events in regenerative therapies are predominantly minor, such as temporary injection-site pain and swelling, with rare severe outcomes [64]. Similarly, Leventoğlu and Böncüoğlu (2024) reported a case of mild, self-limited local reaction following PRP use, emphasizing its generally safe profile despite occasional hypersensitivity [65]. Xiong et al. (2023) further supported PRP’s safety in a meta-analysis of osteoarthritis trials, finding no significant difference in adverse event rates compared to controls, with most events being transient local discomfort [66]. Maisel-Campbell et al. (2020) also found PRP to be well-tolerated in skin aging treatments, with adverse events limited to mild, short-term erythema and edema [67]. Our findings further support the hypothesis that the type and composition of PRP, specifically, the leukocyte content and activation status, are key determinants of tolerability. Leukocyte-rich PRP has been associated with increased levels of catabolic cytokines and metalloproteinases (e.g., IL-1β, MMP-9), leading to synovial inflammation and matrix degradation in both preclinical and clinical models [34, 58]. In contrast, the leukocyte-poor PRP used in this study (1030 ± 130 × 10³/µL, Table 2) minimized leukocyte-derived pro-inflammatory mediators, consistent with the reduced adverse event profile. Additionally, the use of non-activated PRP likely contributes to its safety. Activation with calcium chloride or thrombin triggers immediate platelet α-granule degranulation and fibrin polymerization, potentially inducing a reactive inflammatory environment in confined spaces like tendon sheaths. Non-activated PRP avoids this surge, enabling a physiological, sustained release of bioactive molecules in situ. This mechanistic nuance supports tailoring PRP’s activation status to tissue type and inflammatory burden, particularly for tenosynovitis management [16]. Uniform use of ultrasound guidance during injection further enhanced procedural safety by avoiding iatrogenic tendon trauma, peritendinous leakage, or neurovascular compromise, risks more prevalent with blind injection techniques. Image-guided delivery ensures precise localization of the PRP bolus into the tendon sheath, enhancing both safety and efficacy [68]. Together, these approaches reinforce PRP’s favorable safety profile for tenosynovitis management.

From a clinical perspective, the favorable safety profile of non-activated, leukocyte-poor PRP demonstrated in this trial (4% adverse events in PRP group [*n* = 100], Table 12) broadens its therapeutic potential for chronic tenosynovitis, particularly in patients where corticosteroids are contraindicated, such as those with diabetes, osteoporosis, or risks from repeated exposure. Unlike corticosteroids, which may weaken tendon sheaths or cause systemic side effects, PRP’s autologous, biocompatible nature resulted in no systemic complications, offering a safer alternative for long-term management [69]. In resource-constrained settings, a single ultrasound-guided PRP injection, as employed here, yielded durable reductions in tendon sheath thickness (from 2.8 ± 0.7 mm to 1.9 ± 0.4 mm by Day 21, Table 8) and pain (VAS from 7.3 ± 1.1 to 3.5 ± 0.9, Fig. 3), suggesting cost-effectiveness compared to chronic analgesics or surgical interventions like tenosynovectomy [70]. Furthermore, the absence of serious adverse events supports exploring repeat-dose PRP regimens or adjunctive strategies, such as combining PRP with focused rehabilitation (e.g., tendon gliding exercises) or bracing to enhance functional outcomes (QuickDASH from 58.2 ± 10.5 to 34.5 ± 8.0, Table 8). The biological kinetics observed, particularly the VEGF peak at Day 14 (285.44 ± 50.0 pg/mL, Table 4; Fig. 2), indicate optimal windows for such combination protocols, aligning PRP administration with the proliferative phase of tendon sheath repair to maximize regenerative effects [5]. These findings encourage further investigation into tailored, multimodal PRP-based protocols to optimize tenosynovitis management.

Finally, this trial strengthens the emerging evidence positioning non-activated, leukocyte-poor platelet-rich plasma (PRP) as a disease-modifying therapy for chronic tenosynovitis, beyond mere symptomatic relief. By modulating the local inflammatory milieu (e.g., early PDGF-AB peak at 8 h, 1253.28 ± 160.0 pg/mL, Table 5), stimulating angiogenesis (e.g., VEGF peak at Day 14, 285.44 ± 50.0 pg/mL, Table 4; Fig. 2), and promoting matrix remodeling (e.g., TGF-β suppression by Day 21, 8.07 ± 1.62 ng/mL, Table 7), PRP addresses the underlying pathophysiology of tendon sheath degeneration. These mechanisms contributed to significant reductions in tendon sheath thickness (from 2.8 ± 0.7 mm to 1.9 ± 0.4 mm, Table 8; Fig. 3) and sustained functional improvements (QuickDASH from 58.2 ± 10.5 to 34.5 ± 8.0, Table 8), suggesting structural and regenerative benefits. As trials increasingly incorporate biomarker profiling and growth factor kinetics, personalized PRP protocols, tailored to inflammatory phases or tissue responsiveness, become feasible, supported by the robust platelet enrichment observed (1030 ± 130 × 10³/µL post-PRP, Table 2; Fig. 1). The favorable safety profile (4% adverse events in PRP group [*n* = 100], Table 12), with only mild, transient adverse events, combined with biological efficacy, establishes non-activated PRP as a practical outpatient treatment for chronic tenosynovitis, particularly in resource-constrained settings where cost-effective alternatives to surgery are critical.

The different PRP studies highlight a critical debate in regenerative medicine, where large-scale trials and meta-analyses have yielded mixed conclusions regarding platelet-rich plasma (PRP) superiority over placebo or alternative treatments in chronic tendinopathies, including tenosynovitis. Studies questioning PRP’s efficacy include a 2017 meta-analysis by Fitzpatrick et al. (18 RCTs, *n* ≈ 1,000), which found no significant differences in pain (VAS) or functional outcomes (VISA, DASH) between PRP and saline across various tendinopathies at 3, 6, or 12 months, potentially due to heterogeneity in PRP preparation and tendinopathy subtypes [61]. Similarly, de Jonge et al.’s RCT (*n* = 54, Achilles tendinopathy) reported no significant improvement in VISA-A scores or ultrasonographic tendon structure with PRP compared to saline at 1 year, citing limited sample size and lack of standardized PRP protocols as potential confounders [71]. These findings suggest that variability in PRP composition (e.g., leukocyte-rich vs. leukocyte-poor), activation status, and anatomical differences in tendon pathology may dilute therapeutic effects, particularly in hypovascular tendons like the Achilles [36]. In contrast, studies supporting PRP’s efficacy provide compelling evidence in specific contexts. A 2018 systematic review by Chen et al. (*n* ≥ 1,000) demonstrated significant pain reduction (WMD − 0.84, 95% CI − 1.23 to − 0.44) and functional improvement in lateral epicondylitis and rotator cuff tendinopathy with PRP compared to placebo, particularly with leukocyte-poor formulations [72]. A 2025 meta-analysis by Goulart et al. (*n* ≈ 2,500, rotator cuff tendinopathy) further confirmed PRP’s short-term superiority in pain relief (SMD − 0.45, *p* < 0.01) and functional gains (DASH, Constant scores) over saline, though long-term benefits were less pronounced, potentially due to waning growth factor effects [73]. These positive outcomes align with RCTs showing PRP’s efficacy in recalcitrant tendinopathies as a second-line therapy, particularly when standardized protocols ensure adequate platelet concentrations (> 800 × 10³/µL) [74].

The discrepancies in these findings can be attributed to several factors. First, variability in PRP preparation, leukocyte content, activation status (e.g., calcium chloride vs. non-activated), and platelet dose, leads to inconsistent growth factor release profiles, affecting therapeutic outcomes [13, 61]. Leukocyte-rich PRP, used in some null studies, may increase pro-inflammatory cytokines (e.g., IL-1β, MMP-9), potentially exacerbating synovial inflammation in tenosynovitis [34, 58]. Second, anatomical and pathophysiological differences, such as the hypovascular nature of Achilles tendons versus the synovial environment of tenosynovitis, influence PRP responsiveness, as tendon sheaths may better retain and respond to growth factors like VEGF and PDGF-AB [42, 72]. Third, smaller sample sizes in some negative studies (e.g., *n* = 54 in de Jonge et al.) may lack statistical power to detect moderate effect sizes, particularly for functional outcomes like DASH or WOMAC, which require larger cohorts for robust analysis [71]. Fourth, inconsistent post-injection rehabilitation protocols across trials may confound outcomes, as structured exercise enhances PRP’s regenerative effects by promoting tenocyte mechanotransduction [21].

Our study, with a robust sample size of 200 patients (100 PRP, 100 saline), addresses these limitations through a standardized, non-activated, leukocyte-poor PRP protocol achieving a consistent 4.5-fold platelet enrichment (1030 ± 130 × 10³/µL, Table 2) and rigorous control of rehabilitation compliance. The significant improvements in VAS (− 50.0 mm vs. −31.0 mm, *p* < 0.001), WOMAC (− 35.0 vs. −21.0, *p* < 0.001), DASH (− 35.0 vs. −21.0, *p* < 0.001), and tendon sheath thickness (− 0.9 mm vs. −0.4 mm, *p* < 0.001) at 3 months in the PRP group compared to saline demonstrate clear clinical superiority **[**Tables 8, 10 and 11**]**. These outcomes are mechanistically supported by time-specific growth factor kinetics, with PDGF-AB peaking at 8 h (1253.28 ± 160.0 pg/mL, Table 5) for early inflammation control, EGF at Day 7 (451.84 ± 75.0 pg/mL, Table 6) for proliferation, and VEGF at Day 14 (285.44 ± 50.0 pg/mL, Table 4) for angiogenesis-driven remodeling [Tables 4, 5, 6 and 7]. The strong correlations between platelet counts and VEGF (*r* = 0.62, *p* = 0.001) and PDGF-AB (*r* = 0.58, *p* = 0.002) levels, coupled with their predictive value for tendon thickness reduction (β = −0.18, *p* = 0.021,Tables 9 and 14), provide a biological basis for PRP’s efficacy. The dose-response relationship, with the highest platelet quartile (≥ 1150 × 10³/µL) yielding a 37.2% thickness reduction and 82% responder rate **[**Table 15**]**, further validates the importance of standardized platelet dosing, contrasting with underpowered or heterogeneous studies reporting null effects. Additionally, our focus on chronic tenosynovitis, a synovial condition with distinct vascular and inflammatory dynamics, likely enhances PRP’s efficacy compared to hypovascular tendons, as supported by prior evidence [42].

While acknowledging the validity of null findings in specific contexts, our trial’s large sample size, standardized protocol, and integration of biomarker kinetics with clinical outcomes provide robust evidence for non-activated PRP’s efficacy in chronic tenosynovitis. The alignment of growth factor peaks with healing phases and the dose-dependent effects underscore PRP’s mechanistic plausibility, refuting claims of equivalence to placebo in this context. Future multicenter trials with longer follow-up and diverse PRP formulations are needed to confirm these findings and refine individualized dosing strategies, but our study establishes a strong foundation for PRP as a disease-modifying therapy in regenerative musculoskeletal medicine.

This randomized controlled trial (RCT) is among the first to comprehensively characterize the longitudinal kinetics of VEGF, PDGF-AB, EGF, and TGF-β over 1 month, with clinical outcomes assessed up to 3 months, following non-activated platelet-rich plasma (PRP) therapy compared to saline injection for chronic tenosynovitis. Conducted at Kafrelsheikh University, this study addresses a critical gap in understanding the temporal dynamics of PRP-derived growth factors and their correlation with clinical improvements in pain, function, and tendon sheath remodeling in a Middle Eastern population with a high burden of occupational musculoskeletal disorders [15]. Unlike prior studies limited to short-term growth factor assessments (≤ 7 days) [10, 16], our analysis reveals distinct temporal patterns, early PDGF-AB peaks at 8 h (1253.28 ± 160.0 pg/mL, Table 5), mid-phase EGF elevations at Day 7 (451.84 ± 75.0 pg/mL, Table 6), late VEGF surges at Day 14 (285.44 ± 50.0 pg/mL, Table 4), and TGF-β stabilization, that correlate with superior clinical outcomes in the PRP group (e.g., 50% VAS reduction, 40% WOMAC improvement at 1 month) compared to saline (15% VAS reduction, 10% WOMAC improvement) **[**Tables 10 and 11**]**. By employing a standardized non-activated PRP protocol (1030 ± 130 × 10³/µL, Table 2) and a saline control group, this RCT provides robust evidence for PRP’s efficacy and mechanistic basis, demonstrating its feasibility in resource-constrained settings like Egypt while contributing to global regenerative medicine research.

The temporal dynamics of growth factor release elucidated in this study offer significant clinical implications for optimizing PRP therapy in chronic tenosynovitis. By identifying distinct peaks, PDGF-AB at 8 h for acute inflammation, EGF at Day 7 for proliferation, and VEGF at Day 14 for remodeling, our findings provide a mechanistic framework to time PRP administration for maximal therapeutic benefit, aligning with specific phases of tendon healing [5, 6]. The PRP group’s significant improvements, 57% reduction in pain (VAS: 70.0 ± 15.0 mm to 30.0 ± 12.0 mm) and 42% improvement in function (WOMAC: 60.0 ± 10.0 to 35.0 ± 8.0) at 1 month, sustained at 3 months (VAS: 20.0 ± 10.0 mm, WOMAC: 25.0 ± 7.0), outperformed saline (VAS: 50.0 ± 15.0 mm, WOMAC: 45.0 ± 10.0 at 1 month; *p* < 0.001) **(**Tables 10 and 11**)**, highlighting PRP’s potential as a regenerative alternative to corticosteroids, which risk tendon weakening [1]. The synergy between VEGF and PDGF-AB (β = − 0.18, *p* = 0.021, Table 14) and dose-response relationship with platelet count quartiles (Q4: 37.2 ± 10.5% thickness reduction, Table 15) further underscores PRP’s mechanistic advantages. The observed systemic platelet count increase post-injection (21.2% rise, 277.68 ± 61.93 × 10³/µL) is exploratory, as prior studies prioritize PRP concentrate counts (1030 ± 130 × 10³/µL, 4.5× enrichment, Table 2) for therapeutic efficacy [13, 16]. Conducted in Egypt, this study demonstrates the scalability of PRP in resource-constrained settings, offering a model for clinical practice in the Middle East and beyond. However, future studies are needed to validate these findings.

This study has several limitations that warrant consideration. First, the sample size (*n* = 200, 100 PRP, 100 saline), while powered to detect significant between-group differences (e.g., VAS reduction ≥ 20 mm), may limit generalizability to broader populations with chronic tenosynovitis. Second, as a single-center study, site-specific factors, such as patient demographics or clinical practices at Kafrelsheikh University, may introduce bias. Third, while the 3-month follow-up captured sustained clinical outcomes, longer-term studies are needed to assess the durability of pain relief and tendon remodeling. Fourth, the focus on non-activated, leukocyte-poor PRP may not be generalized to activated or leukocyte-rich formulations, which warrants a separate investigation [13]. Fifth, the study’s focus on upper limb tenosynovitis (e.g., De Quervain’s, trigger finger, flexor tenosynovitis) may not extend to lower limb conditions, such as Achilles tenosynovitis, warranting further research to evaluate PRP efficacy in other anatomical sites. These limitations are mitigated by the study’s rigorous RCT design, standardized PRP protocol, blinded assessments, and robust statistical analyses, but future research should incorporate larger, multicenter trials with extended follow-up and diverse PRP preparations to validate and expand upon these findings.

## Conclusion

This randomized trial demonstrates that non-activated, leukocyte-poor PRP yields distinct, time-dependent growth factor peaks, most notably PDGF-AB at 8 h and VEGF at Day 14, that align with clinically meaningful improvements in pain, function, and tendon structure in chronic tenosynovitis. The observed dose–response relationship supports the integration of platelet concentration thresholds into clinical protocols and regulatory frameworks, enabling more consistent and effective PRP use. Autologous PRP demonstrated a favorable safety profile, underscoring its suitability as a viable outpatient intervention in both high-resource and resource-constrained settings. These findings provide a mechanistic foundation and practical roadmap for personalized, biomarker-guided PRP therapy in regenerative musculoskeletal medicine.

## Supplementary Information

Supplementary Material 1.

## Data Availability

All relevant data are included in this published article.
